# DNA polymorphism and selection at the *bindin* locus in three *Strongylocentrotus* sp. (Echinoidea)

**DOI:** 10.1186/s12863-016-0374-5

**Published:** 2016-05-12

**Authors:** Evgeniy S. Balakirev, Maria Anisimova, Vladimir A. Pavlyuchkov, Francisco J. Ayala

**Affiliations:** A. V. Zhirmunsky Institute of Marine Biology, Far Eastern Branch of the Russian Academy of Science, Vladivostok, 690041 Russia; Department of Ecology and Evolutionary Biology, University of California, 321 Steinhaus Hall, Irvine, CA 92697-2525 USA; Far Eastern Federal University, Vladivostok, 690950 Russia; Institute of Applied Simulation, School of Life Sciences and Facility Management, Zürich University of Applied Sciences, Wädenswil, 8820 Switzerland; Swiss Institute of Bioinformatics, Lausanne, 1015 Switzerland; Pacific Research Fisheries Centre (TINRO-Centre), Vladivostok, 690600 Russia

## Abstract

**Background:**

The sperm gene *bindin* encodes a gamete recognition protein, which plays an important role in conspecific fertilization and reproductive isolation of sea urchins. Molecular evolution of the gene has been extensively investigated with the attention focused on the protein coding regions. Intron evolution has been investigated to a much lesser extent. We have studied nucleotide variability in the complete *bindin* locus, including two exons and one intron, in the sea urchin *Strongylocentrotus intermedius* represented by two morphological forms. We have also analyzed all available *bindin* sequences for two other sea urchin species, *S. pallidus* and *S. droebachiensis*.

**Results:**

The results show that the *bindin* sequences from the two forms of *S. intermedius* are intermingled with no evidence of genetic divergence; however, the forms exhibit slightly different patterns in *bindin* variability. The level of the *bindin* nucleotide diversity is close for *S. intermedius* and *S. droebachiensis*, but noticeably higher for *S. pallidus*. The distribution of variability is non-uniform along the gene; however there are striking similarities among the species, indicating similar evolutionary trends in this gene engaged in reproductive function. The patterns of nucleotide variability and divergence are radically different in the *bindin* coding and intron regions. Positive selection is detected in the *bindin* coding region. The neutrality tests as well as the maximum likelihood approaches suggest the action of diversifying selection in the *bindin* intron.

**Conclusions:**

Significant deviation from neutrality has been detected in the *bindin* coding region and suggested in the intron, indicating the possible functional importance of the *bindin* intron variability. To clarify the question concerning possible involvement of diversifying selection in the *bindin* intron evolution more data combining population genetic and functional approaches are necessary.

**Electronic supplementary material:**

The online version of this article (doi:10.1186/s12863-016-0374-5) contains supplementary material, which is available to authorized users.

## Background

*Bindin* is one of the most thoroughly investigated genes in sea urchins (reviews in [1–8]). The gene is expressed in males during spermatogenesis [9, 10] and encodes a sperm protein mediating species-specific gamete adhesion and membrane fusion during sea urchin fertilization. Bindin recognizes two different egg surface species-specific sperm receptors, 350-kDa and EBR1 [1, 8]. The bindin protein is the main content of the sea urchin sperm acrosomal vesicles and it serves as molecular glue connecting the sperm to the egg [8, 11]. It has been suggested that bindin plays an important role in post-mating pre-zygotic isolation accounting for reproductive isolation between sea urchin species [3, 5–8, 12, 13]. Indeed, reproductive success has been shown to correlate with the level of *bindin* sequence divergence [14–16]. Zigler et al. [17] detected a clear correlation between nonsynonymous *bindin* divergence and average gametic incompatibility in 14 sea urchin species pairs; however, no such correlation was found between mitochondrial divergence and gamete compatibility [17, 18].

The mature bindin protein is highly variable in length, ranging from 193 to 418 amino acids among ten sea urchin genera from which it has been sequenced [3, 4]. Comparisons of DNA sequences from ten sea urchin species, belonging to six orders, have revealed the presence of a central region in which 55–60 amino acids are highly conserved (the “core” region), flanked by two variable regions [3, 4]. The conserved fusogenic “B18” region has remained unchanged over the entire 250 million year span of extant echinoid evolution [3, 4]. It includes a stretch of 18 amino acids involved in membrane fusion [19]. Sea stars and sea urchins, which diverged roughly 450–500 million years ago, have just one amino acid difference within the B18 region [20]. The flanking bindin repeats, which vary in length among different species, bestow a species recognition mechanism [21].

The *bindin* gene is variable not only structurally but also in the pattern of evolution. In some species the evolution of the gene does not deviate from neutrality; however, in other cases strong positive selection was apparent (review in [4–7]). Adaptive divergence of the *bindin* coding region was detected in four sea urchin genera, *Echinometra* [22–24], *Strongylocentrotus* [25, 26], *Heliocidaris* [27], and *Paracentrotus* [28, 29], but not in four other genera, *Arbacia* [30, 31], *Tripneustes* [32], *Lytechinus* [33], and *Mesocentrotus* [34]. Positive selection on *bindin* variation was detected also in the sea stars *Patiria miniata* [35] and *Pisaster ochraceus* [36], but not in *Pisaster brevispinus*, which is consistent with greater polyspermy harm in *P. ochraceus* [36].

A number of hypotheses explaining specific bindin evolution in different sea urchin genera have been suggested, including reinforcement [37] (selection for prezygotic isolation to prevent heterospecific fertilization and avoid production of inferior hybrids), sexual selection at the cellular level [14, 38] (evolution of reproductive traits including “cryptic female choice”), sexual conflict [39, 40] (balance between sperm competition and egg polyspermy avoidance), and immunological defense [41] (antagonistic coevolution with pathogens). All hypotheses are based on the analysis of the bindin protein-coding region (without consideration the intron) (for details see [2, 4–7, 13]).

Since the time of bindin discovery as a critical protein mediating species-specific gamete recognition and fertilization in sea urchins (review in [8]), a wealth of data have been obtained concerning the molecular evolution of this protein (as well as other proteins involved in reproduction). This group of proteins was considered one of the most convincing examples of evolution driven by positive selection (review in [5, 7, 13]). However, the mechanisms that drive adaptive diversification of reproductive proteins remain largely unknown, as pointed out by Vacquier and Swanson [7] (page 14): “The factors responsible for such strong selection on fertilization proteins are at present difficult to define with certainty”. No noticeable divergence in bindin was detected between the sympatric sea urchin species *Pseudoboletia indiana* and *P. maculata* [42], or the subspecies *Heliocidaris erythrogramma erythrogramma* and *H. e. armigera* [43], which hybridize extensively [42, 43]. The data of Addison and Pogson [44] on asymmetric introgression among strongylocentrotid sea urchins demonstrates that gamete traits alone cannot be responsible for maintaining species integrities; and that genetic boundaries between strongylocentrotid sea urchin species in the northeast Pacific appear to be related to postzygotic isolating mechanisms, which were consistently associated with divergence times, but not with intrinsic gametic incompatibilities *per se*. These observations challenge a generally accepted doctrine that considers that gamete recognition proteins are the critical factor for developing and maintaining species boundaries, and for the evolution of gamete incompatibility in spawning marine invertebrates (see references above).

The vast majority of the *bindin* gene studies have focused on coding sequence variation and/or divergence. The intron sequences usually were not investigated at all or only superficially. Ignoring the intron sequences is surprising, even from the structural point of view. The *bindin* gene of strongylocentrotid sea urchins has a single intron about 0.9 kb in length (ranging from 908 to 965 bp in different individuals and species), and two exons of the mature bindin (around 0.5 kb; ranging from 507 to 591 bp in different individuals and species). The *bindin* intron may contain important regulatory sites as it has been shown for many other introns (e.g., [45]) and represent a rich source of adaptively important variation (e.g., [46, 47]). Consequently, we have investigated *bindin* nucleotide polymorphism and divergence in both, the coding region and the intron in *Strongylocentrotus* sea urchins, the model group used for studying the evolution of reproductive proteins [7].

Three congeneric sea urchin species, *Strongylocentrotus intermedius* (A. Agassiz, 1863), *S. pallidus* (G. O. Sars, 1871), and *S. droebachiensis* (O. F. Müller, 1776) represent a monophyletic clade within the family Strongylocentrotidae [48]. The species are common in the North Atlantic, Arctic, and Pacific continental regions [49, 50]. The *Strongylocentrotus* sea urchins are important farmed and harvested species in many countries including Japan, China, North and South Korea, Norway, Russia, Canada, and U.S.A. [50]. The most widely distributed species, *S. pallidus*, inhabits the North Atlantic, Arctic, and Pacific continental shelves and slopes, with highest abundances at depths of 150–300 m and up to 1600 m [49]. *S. droebachiensis* has somewhat similar but not so wide geographical distribution as *S. pallidus*, preferring littoral waters, with highest abundances at 5–10 m of depth. These two species typically have clearly different depth distribution [49, 51, 52]. In shallow areas, however, *S. pallidus* and *S. droebachiensis* may occur sympatrically [49, 53]. The distribution of *S. intermedius* is limited to the northwest Pacific region, including the Sea of Japan, the Sea of Okhotsk and the East coast of Kamchatka, the Southern Kuril Islands, and the coast of Japan [49, 50]. This species mostly occurs from the littoral and upper sublittoral zone down to a depth of 25 m. The northern Primorye (Sea of Japan) populations of *S. intermedius* consist of two sympatric morphological forms, “usual” (U) and “gray” (G). The two forms are different in morphology and preferred bathymetric distribution. We have shown that these two forms predominantly harbored highly divergent bacterial symbiont lineages, although they were not distinguished genetically [54]. The geographical ranges of *S. intermedius*, *S. pallidus*, and *S. droebachiensis* overlap in the eastern Sakhalin and Kuril islands, and in the western coast of the Sea of Japan. Thus, all three species can occur sympatrically in some parts of their geographic distribution. However, direct evidence for mixing settlements is not available, and differences in ecological preferences may make the mixing unlikely. Nevertheless, Addison and Hart [55] detected in mitochondrial DNA significant evidence of introgression between *S. pallidus* and *S. droebachiensis*; which was later confirmed with four nuclear loci [44].

The purpose of the present study is to investigate the patterns of nucleotide variability in the complete *bindin* locus, including the two exons and one intron of *S. intermedius* (represented by two morphological forms) from the North Primorye population (the Sea of Japan). For comparative purposes, we have analyzed the *bindin* sequences of two congeners, *S. pallidus* and *S. droebachiensis*, obtained from GenBank. We have detected very different patterns of nucleotide variability and divergence in the coding and intron regions, but with striking similarity among all three species studied. A clear signal of positive selection was detected in the coding region; neutrality tests as well as maximum likelihood analyses suggest the action of diversifying selection in the *bindin* intron. We have also analyzed the two morphological forms of *S. intermedius* separately and found only slight differences among them concerning the patterns of *bindin* variability.

## Methods

### Sea urchin samples and sequences

A sample of *S. intermedius* (25 individuals) was obtained from the sea urchin settlement close to Cape Zolotoi (46°15’086”N, 138°06’646”E; Sea of Japan, Pacific Ocean). The U (12 individuals) and G (13 individuals) forms were collected at depths of 5 to 10 m and 15 to 20 m, respectively. The procedures for DNA extraction, amplification, cloning, and sequencing have been described previously [47, 56, 57]. A 1,809 bp fragment of the nuclear gene *bindin* was amplified using primers: 5′-tctgacgattcgaaaagaggag-3′ (forward primer) and 5′-attagcgtctatatctagttag-3′ (reverse primer). The alignment of the *bindin* sequences of *S. franciscanus* (M59490; [58]) and *S. purpuratus* (M14487; [59]) was used to design the primers. The amplified fragments include the complete *bindin* coding region (285 codons without counting the terminating stop codon) consisting of exon I (237 bp), intron (951 bp), and exon II (621 bp) that encompass the complete mature bindin protein. The 273-bp repeat region of exon II containing a 21-bp repeat motif was excluded from the analysis because orthology/paralogy relationships are uncertain in these sequences. Twelve *bindin* sequences are from Balakirev et al. [54] (EU003202-EU003213). A 1,056-bp fragment of the mitochondrial gene encoding cytochrome c oxidase subunit 1 (*COI*) was amplified in 59 individuals of *S. intermedius* using the following primers: 5′-acactttatttgatttttgg-3′ (forward) and 5′-cccattgaaagaacgtagtgaaagtg-3′ (reverse) [60]. These sequences include the mitochondrial DNA region covering 352 codons of the *COI* gene, corresponding to positions 5854 to 6909 in the complete *S. purpuratus* mitochondrial sequence [61]. The new sequences have been deposited in GenBank under accession numbers KP774723—KP774781 (*COI*) and KP774782—KP774794 (*bindin*). (See Additional file 1: Text S1 for PCR details and Text S2 for the *bindin* sequences of the genus *Strongylocentrotus* and close species obtained from the GenBank database.)

### DNA sequence analysis

The *bindin* sequences were assembled using the program SeqMan (Lasergene, DNASTAR, Inc.). Multiple sequence alignment was carried out using CLUSTAL W [62]. DnaSP, v. 5 [63] and PROSEQ, v. 2.9 [64] were used to analyze the data by the “sliding window” method [65], and for most intraspecific analyses; MEGA, v. 5 [66] was used for basic phylogenetic analyses (see [57, 67]). Departures from neutral expectations were investigated using the tests HKA [68], Tajima’s [69], McDonald and Kreitman’s [70], Fu and Li’s [71], Hudson’s et al. [72], McDonald’s [73, 74], Kelly’s [75], Depaulis and Veuille’s [76], and Wall’s [77]. The HKA test was used to compare each *bindin* exon to the other, or compare the exons to the intron (the mitochondrial *COI* gene is not appropriate to use for the HKA test as a reference sequence to assess the deviation from neutrality in the nuclear *bindin* gene). The permutation approach of Hudson et al. [78] was used to estimate the significance of sequence differences between the morphological forms. Simulations based on the coalescent process with or without recombination [79–81] were performed with the DnaSP and PROSEQ programs to estimate the probabilities of the observed values of Tajima’s *D*, Kelly’s *Z*_*nS*_ and Wall’s *B* and *Q* statistics and confidence intervals of the nucleotide diversity values. Simulations with 10,000 replicates were conditional on the sample size, the observed number of segregating sites, and the alignment length, with the population recombination rate parameter, *ρ* (or 4*N*_0_*r*) set to the gene estimates. The method of Sawyer [82] was used to detect gene conversion events. The population recombination rate was analyzed with the permutation-based approach [83]. The alignments were also analyzed for evidence of recombination using various recombination detection methods implemented in the program RDP3 [84].

### Codon-based sequence analyses

Probabilistic Markov codon-substitution models were fitted to coding alignments assuming phylogenetic trees reconstructed by the maximum likelihood (ML), under model LG + Г + F using PhyML v.3 [85]. Model parameters were estimated using ML. These models measure selective pressure using the ratio of nonsynonymous to synonymous substitution rates ω = *d*_N_/*d*_S_, which may vary among sites. Positive or negative selection is evidenced by significant deviations of the ω-ratio from 1. We used models that assume constant synonymous rates M0, M3, M7, M8 [86] and FMutSel0, FMutSel [87] as implemented in PAML v. 4 [88], and a model accounting for variability of synonymous rates over sites GYxHKY Dual GDD 3x3 [89], later referred as M3-Dual, and implemented in HYPHY [90]. Hypotheses concerning selection, codon bias, and rate variability were tested using likelihood ratio tests (LRTs). For a review about the application of codon models, see Anisimova and Kosiol [91]. Models combining coding and noncoding sequences were used to test for positive selection on noncoding regions, as implemented in EvoNC [92]. The strength of selection on noncoding regions was measured by ζ, the ratio of the substitution rate in noncoding regions relative to the synonymous rate in coding regions. Under neutrality, these rates are expected to be similar (ζ ≈ 1). Significant deviations from 1 may be considered to be evidence of positive (ζ > 1) or negative (ζ < 1) selection on noncoding regions. Consequently, the null model allowed two classes of sites in noncoding regions: a neutral class with ζ = 1 and a class of sites evolving under negative selection where the average exonic synonymous rate was higher than the substitution rate in the noncoding regions (ζ < 1). The alternative model also allowed two classes of sites, but the rate ratio was estimated for both classes under constraints: ζ ≥ 1 for positive and neutral selection class, and ζ < 1 for the negatively selected class. A Bayesian approach was used to predict sites affected by positive selection in both coding and noncoding regions [86, 89, 92, 93].

## Results and Discussion

### Nucleotide diversity

**Fig. 1:**
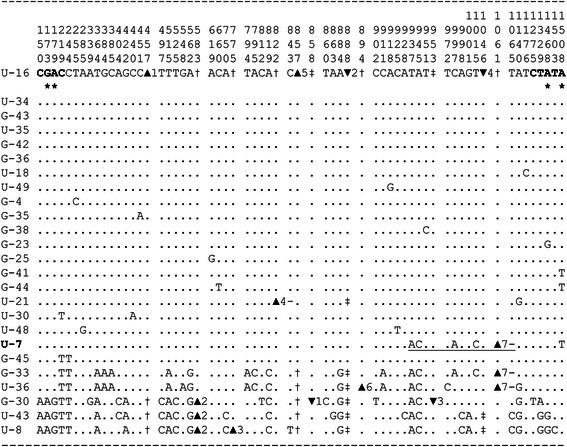
DNA polymorphism in the *bindin* gene of the sea urchin *Strongylocentrotu*s *intermedius*. The numbers above the top sequence represent the position of segregating sites and the start of a deletion or insertion. Nucleotides are numbered from the beginning of our sequence (position 877 in [59], starting the mature bindin protein). The coding nucleotides are in bold. Amino acid replacement polymorphisms are marked with asterisks. Dots indicate the same nucleotide as the reference sequence. The hyphens represent deleted nucleotides. ▲ denotes a deletion; † denotes the absence of a deletion; ▼ denotes an insertion; ‡ denotes the absence of an insertion. The recombinant sequence U-7 is in bold; the putative conversion tract is underlined. The exon - intron coordinates are: 1–237: exon I; 238–1191: intron; 1192–1518: exon II

Figure 1 shows all 54 polymorphic sites in our sample of 25 sequences of the *S. intermedius bindin* gene excluding a repeat region in exon II (Additional file 2: Figure S1 and Text S3 represent polymorphism characteristics in a repeat region). The *bindin* polymorphic sites of *S. pallidus* and *S. droebachiensis* are presented in Additional file 2: Figures S2 and S3, respectively. There are eleven length polymorphisms in the intron region (nine deletions and two insertions; see Additional file 2: Text S4 for details); the intron region harbors most length polymorphisms also in *S. pallidus* and *S. droebachiensis*. A particularly interesting long insertion (539 bp) was detected in the intron of *S. droebachiensis*. The insertion is flanked by an 18-bp inverted repeat sequence (ttaaaggtactatgtccc) and it may represent a transposable element. It has been shown that the oyster *bindin* gene contains a 3.6 kb retroposon [94].

Figure 1 indicates strong haplotype structure for the *bindin* gene in *S. intermedius*. There is a set of nineteen sequences, U-16, U-34, G-43, U-35, G-42, G-36, U-18, U-49, G-4, G-35, G-38, G-23, G-25, G-41, G-44, U-21, U-30, U-48, and G-45 that are very similar to each other and differ by a fixed single nucleotide deletion (position 857) and five synonymous and intronic substitutions from a second set of five sequences, G-33, U-36, G-30, U-43, and U-8. Moreover, three out of four replacement substitutions (positions 173, 179, and 1508) segregate within each set of sequences but not between them (Fig. 1). Strong haplotype structure is also detected for the two other *Strongylocentrotus* species, *S. pallidus* and *S. droebachiensis* (Additional file 2: Figures S2 and S3).

Highly divergent haplogroups were frequently observed in many genes of *Arabidopsis* (e.g., [95] and references therein), *Drosophila* (e.g., [46, 47, 96–99]) and other eukaryotes including sea urchins [22–24, 26–29]; they may represent a signature of demographic and/or adaptive processes (e.g., [72, 100–102]). The pattern of variation observed in the genes with dimorphic haplotype structure would seem to be compatible with a constant-size neutral process with no recombination [101, 102]. Indeed, recombination is low for the *bindin* locus. The method of Hudson and Kaplan [103] reveals a minimum of three recombination events in the *bindin* region analyzed in *S. intermedius* and *S. droebachiensis*, but none in *S. pallidus*. Statistically significant signals of recombination are detected by seven methods implemented in the program RDP3 [84] in *S. intermedius* and *S. pallidus*, but not in *S. droebachiensis* (Additional file 3: Table S1). The population recombination rate (*ρ* = 4*N*_*e*_*r*, where *N*_*e*_ is the effective population size and *r* is the recombination rate/nucleotide site/generation) obtained by the coalescent-based method of McVean et al. [83] is 0.0047, 0.0005, and zero for *S. intermedius*, *S. pallidus*, and *S. droebachiensis*, respectively (Additional file 3: Table S2). Low levels of recombination had been also reported for the *bindin* gene of sea urchins *Echinometra* [24], *Strongylocentrotus* [26], *Heliocidaris* [27], and *Paracentrotus* [28]. Thus, recombination is present within the *bindin* gene of the strongylocentrotid sea urchins; however, the frequency of recombination events is less pronounced in comparison, for instance, with some *Drosophila* genes [46, 47, 97, 98] or sperm *bindin* in oyster [104].

The patterns of haplotype structure and low recombination are consistent with the strong linkage disequilibrium (LD) within the *bindin* gene in all three sea urchin species. There are 66.4, 45.1, and 47.1 % significant associations between the informative sites (Fisher’s exact test) for *S. intermedius*, *S. pallidus*, and *S. droebachiensis*, respectively. After the Bonferroni correction, 8.3 and 15.0 % associations remain significant for *S. intermedius* and *S. droebachiensis*; none is significant for *S. pallidus*. The distribution of the LD values is non-uniform along *bindin* (Additional file 4: Figure S4; see below the subsection “Sliding window analysis”). Significant associations are mostly due to polymorphic sites located within the *bindin* exon I and intron in *S. intermedius*; there is also a clear peak in exon II of *S. pallidus* and *S. droebachiensis* (Additional file 4: Figure S4). LD is more pronounced in the *S. intermedius* U form (14.2 % significant associations with Fisher exact test) than in the G form (5.4 % significant associations).

**Fig. 2:**
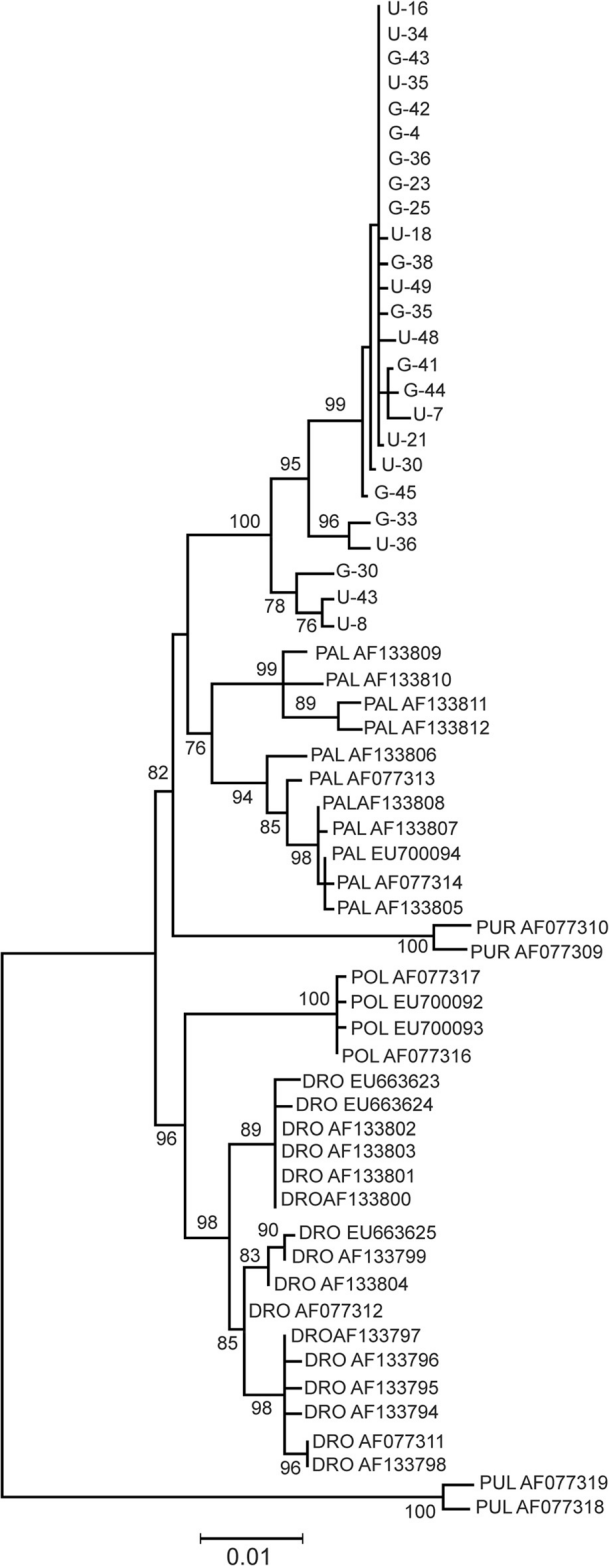
Maximum likelihood tree of the strongylocentrotid sea urchins *bindin* sequences. The tree is based on Kimura 2-parameter (K2P) model as the best-fitting model of substitution under the maximum likelihood criterion [66] for constructing an ML tree of the *bindin* sequences. The numbers at the nodes are bootstrap percent probability values based on 1,000 replications. The *bindin* sequences of *Hemicentrotus pulcherrimus* (AF077318 and AF077319) are used as outgroups. The specimens of *S. intermedius* are marked with letters “G” and “U”. DRO = *S. droebachiensis*, PAL = *S. pallidus*; POL = *S. polyacanthus*; PUR = *S. purpuratus*; PUL = *Hemicentrotus pulcherrimus*

Figure 2 displays a maximum likelihood (ML) tree of the *bindin* sequences obtained for the *S. intermedius* forms in the present study, along with other *bindin* sequences from the strongylocentrotid sea urchins obtained from GenBank. Since the *bindin* coding region of the *Strongylocentrotus* sea urchins is under positive selection (see section “Background”), this tree is not a good reflection of the phylogeny of related species, but it serves to show the genetic structure of the data. Highly structured patterns of the *bindin* gene variation (Fig. 1, Additional file 2: Figures S2 and S3) are reflected in the ML tree: different sets of sequences (described above) form separate clusters within *S. intermedius*, *S. pallidus*, and *S. droebachiensis* with significant bootstrap support. The U and G forms of *S. intermedius*, however, do not form separate clusters: the tree shows the *bindin* sequences from the two forms are intermingled with no evidence of genetic divergence (for *bindin*: *F*_st_ = − 0.0291, *P* = 0.6342; total sequence divergence between the forms *D*_xy_ = 0.0064; for *COI*: *F*_st_ = − 0.0570, *P* = 0.7587; *D*_xy_ = 0.0047). These data are in accordance with our previous results [54], confirming that the U and G morphological forms of *S. intermedius* are not distinct biological species.

Table 1 shows estimates of nucleotide polymorphism and divergence for the entire *S. intermedius* data set as well as for other strongylocentrotid sea urchins obtained from GenBank. The overall mean divergence between species for the *bindin* gene (calculated with all available sequences, Fig. 2) is 0.0244 ± 0.0023, which is lower than for the *COI* gene, 0.0415 ± 0.0030. Divergence is not uniform across the *bindin* functional regions and site classes. It is higher in synonymous sites of both exons than in intron sites (Table 1). The total *bindin* variability is low for *S. intermedius* (π = 0.0060 ± 0.0010) and *S. droebachiensis* (π = 0.0081 ± 0.0015) but higher in *S. pallidus* (π = 0.0158 ± 0.0021). The same trend is observed for the silent variability (Table 1). The difference is mostly due to the intron, which is more variable in *S. pallidus* than in *S. droebachiensis* and *S. intermedius*: π = 0.0205 ± 0.0031 versus 0.0080 ± 0.0018 and 0.0078 ± 0.0016. The comparison of the U and G morphological forms of *S. intermedius* (excluding the recombinant sequence U-7) shows that the total *bindin* variability of the U form (π = 0.0076 ± 0.0013) is 1.6 times higher than in the G form (π = 0.0048 ± 0.0008), a marginally significant difference (*P* = 0.05) in coalescent simulations using parsimony informative polymorphic sites with the population recombination rate 0.005 obtained by the method of McVean et al. [83]. There are no variability differences in the mitochondrial *COI* gene between the two forms; the total variability is 0.0047 for the U form and 0.0048 for the G forms.

**Table 1:** Nucleotide diversity and divergence in the *bindin* gene of the sea urchins *Strongylocentrotus intermedius*, *S. pallidus*, and *S. droebachiensis*

	Exon I			Exon II			Exon I + Exon II			Intron	Silent	Total
	Syn	Nsyn	Total	Syn	Nsyn	Total	Syn	Nsyn	Total			
INT (25)												
N	57	180	237	78	246	324	135	426	561	871	1009	1435
S	2	2	4	3	2	5	5	4	9	39	44	48
π	0.0111	0.0025	0.0046	0.0050	0.0018	0.0026	0.0076	0.0021	0.0034	0.0078	0.0077	0.0060
θ	0.0092	0.0030	0.0045	0.0136	0.0022	0.0050	0.0117	0.0025	0.0047	0.0119	0.0118	0.0090
*K*_int-pul_	0.1925	0.	0.1165	0.1465	0.	0.0702	0.1654	0.	0.0892	0.0739	0.0851	0.0796
PAL (11)												
N	57	180	237	75	234	309	132	414	546	934	1069	1483
S	4	1	5	1	4	5	5	5	10	53	58	63
π	0.0271	0.0046	0.0100	0.0022	0.0073	0.0061	0.0131	0.0061	0.0078	0.0205	0.0195	0.0158
θ	0.0253	n.a.	0.0072	0.0046	0.0058	0.0055	0.0131	n.a.	0.0063	0.0194	n.a.	0.0145
*K*_pal-pul_	0.1408	0.	0.1042	0.1704	0.	0.0755	0.1569	0.	0.0883	0.0736	0.0825	0.0786
DRO (16)												
N	56	180	237	75	240	315	131	420	552	919	1054	1474
S	1	3	4	4	4	8	5	7	11	27	32	38
π	0.0041	0.0062	0.0057	0.0192	0.0077	0.0105	0.0128	0.0071	0.0084	0.0080	0.0086	0.0081
θ	0.0053	0.0050	0.0051	0.0160	0.0050	0.0077	0.0114	0.0050	0.0066	0.0089	0.0092	0.0080
*K*_dro-pul_	0.1790	0.	0.1042	0.1630	0.	0.0773	0.1699	0.	0.0890	0.0671	0.0784	0.0748

The region analyzed represents the full mature *bindin* gene, which includes exon I (excluding the signal peptide), intron, and exon II (excluding the repeat region)

INT = *S. intermedius*, DRO = *S. droebachiensis*, PAL = *S. pallidus*; N, number of sites (indels are excluded); S, number of polymorphic sites; π, average number of nucleotide differences per site among all pairs of sequences ([134], p. 256), obtained for the silent, synonymous, nonsynonymous, and total number of sites; θ, average number of segregating nucleotide sites among all sequences, based on the expected distribution of neutral variants in a panmictic population at equilibrium [135]; *K*_int-pul_, *K*_pal-pul_, and *K*_dro-pul_ are the average proportion of nucleotide differences between *S. intermedius*, *S. pallidus*, *S. droebachiensis* and *Hemicentrotus pulcherrimus* (AF077318 and AF077319), respectively, corrected according to [136]; Syn, synonymous sites; Nsyn, nonsynonymous sites; Silent, silent sites (synonymous and noncoding intronic sites). The segregating sites associated with indels are excluded from the π, θ, and, *K* calculations

The nucleotide variability and divergence detected in the *bindin* gene of *Strongylocentrotus* species is in the range of values observed in other genes involved in reproduction (reviews in [5, 7]). The silent variability of *bindin* in *S. pallidus* (π = 0.0195) is close to that of one of the most polymorphic genes of *Drosophila melanogaster*, ψ*Est-6* (π = 0.0253) [97]. Extraordinary high level of intraspecific diversity has also been detected in oyster sperm *bindin* [104].

### Sliding window analysis

**Fig. 3:**
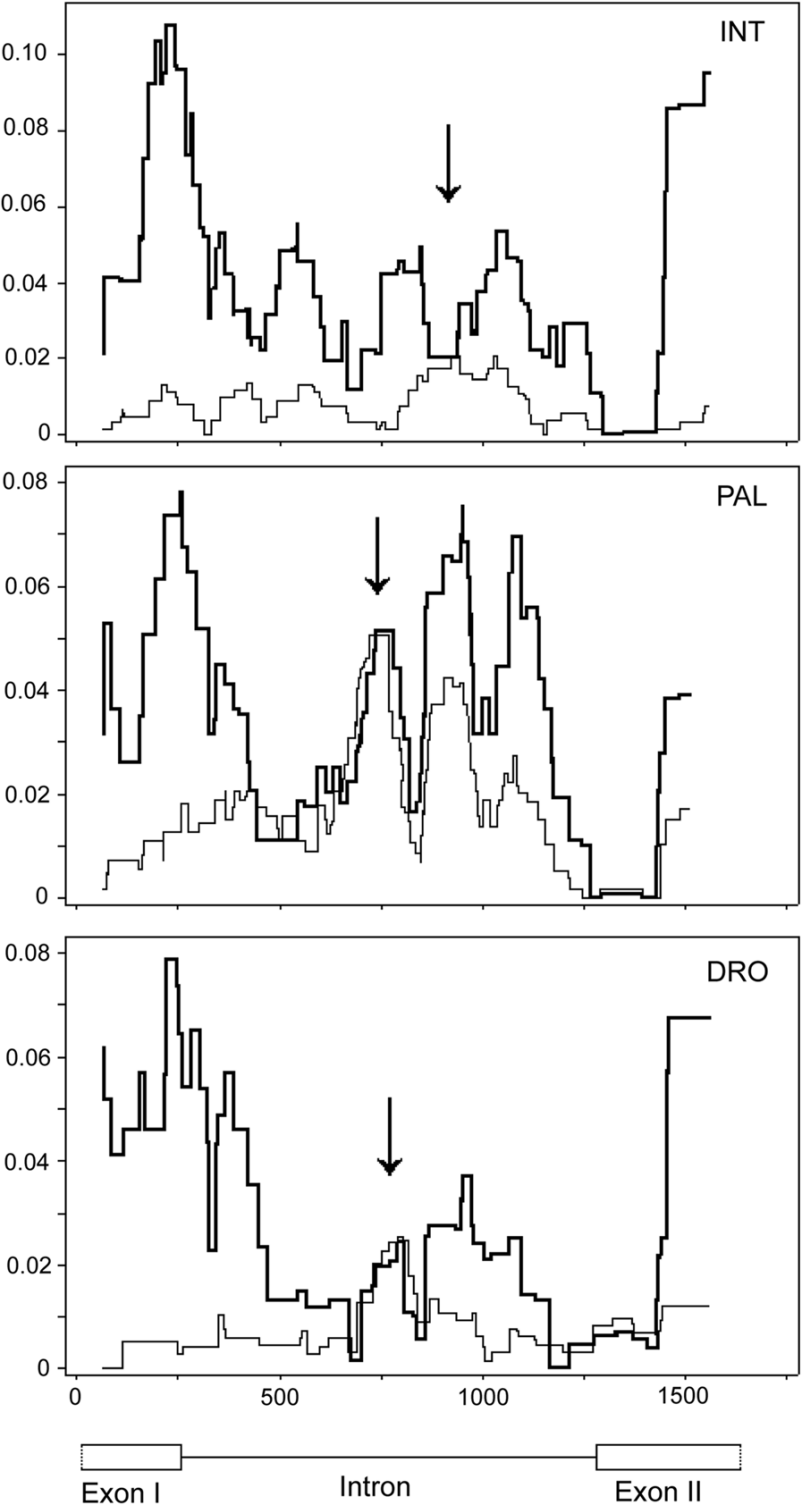
Sliding-window plots of polymorphism (*π*, thin line) and divergence (*K*, thick line) along the *bindin* gene of *S. intermedius* (INT), *S. pallidus* (PAL), and *S. droebachiensis* (DRO). The *bindin* sequence of *S. polyacanthus* (AF077317) was used for the *K* calculations. Window sizes are 100 nucleotides with 1-nucleotide increments. A schematic representation of the *bindin* gene is at the bottom. Vertical arrows indicate the locations of the regions with high within species polymorphism and low between species divergence

The distribution of polymorphism and divergence along the *bindin* gene is non-uniform and has striking similarity in the three sea urchin species (Fig. 3). Nucleotide polymorphism is much lower than divergence in exons but in some regions it is close to the level of divergence in the intron (indicated by vertical arrows). These intron locations are possible targets of diversifying selection [65, 105], which is supported by the neutrality tests and the maximum likelihood analysis (see below).

We have compared the patterns of polymorphism in the three sea urchin species by calculating correlations of this estimate from sliding windows over the *bindin* gene (Fig. 3). To obtain equal number of windows for all species we excluded indels and the *S. intermedius* sequence U-21 with a 48-bp deletion in intron. We also excluded the recombinant sequences in *S. intermedius* (U-7; Fig. 1) and *S. pallidus* (PAL_AF133806 and PAL_AF077313; Additional file 2: Figure S2). The sliding window sizes were 100 nucleotides with 25-nucleotide increments, which produced 52 windows along the *bindin* gene used for the correlation analysis.

We have found significant correlations of polymorphism patterns between the *S. intermedius*—*S. pallidus* (Spearman’s coefficient of rank correlation, rho = 0.374; *P* = 0.0062) and *S. droebachiensis*—*S. pallidus* (rho = 0.441; *P* = 0.0011) species pairs. No correlation was however detected for the *S. intermedius*—*S. droebachiensis* (rho = − 0.043; *P* = 0.7606) species pair. *S. intermedius* and *S. droebachiensis* are the most diverged species among five sea urchins belonging to the genus *Strongylocentrotus* (Fig. 2). An absence of correlation in polymorphism patterns between these two species might be explained by the fact that the pronounced intron peak of polymorphism in *S. intermedius* occurs in slightly shifted coordinates (approximately on 185 bp) in comparison with *S. pallidus* and *S. droebachiensis* (this peak is marked by a vertical arrow on Fig. 3).

We have also compared the patterns of divergence along the *bindin* gene in the three sea urchin species (Fig. 3) by the same approach as above. The *bindin* sequence of *S. polyacanthus* (AF077317) was used in interspecific comparisons. The correlations of divergence patterns between all three species are highly significant: *S. intermedius*—*S. droebachiensis* (rho = 0.719; *P* < 0.0001), *S. intermedius*—*S. pallidus* (rho = 0.657; *P* < 0.0001), and *S. droebachiensis*—*S. pallidus* (rho = 0.748; *P* < 0.0001).

Thus, significant correlations between the patterns of nucleotide diversity in different species support the suggestion that the *bindin* gene evolves similarly (but not identically) in *S. intermedius*, *S. droebachiensis*, and *S. pallidus*. The evolutionary vectors are remarkably similar for these sea urchin species that diverged around 3–7 million years ago [48]. The revealed pattern is consistent with the phenomenon of parallel evolution, which results from similar or identical mutations maximizing adaptation in independent evolutionary lineages (review in [106]).

### Tests of neutrality

**Fig. 4:**
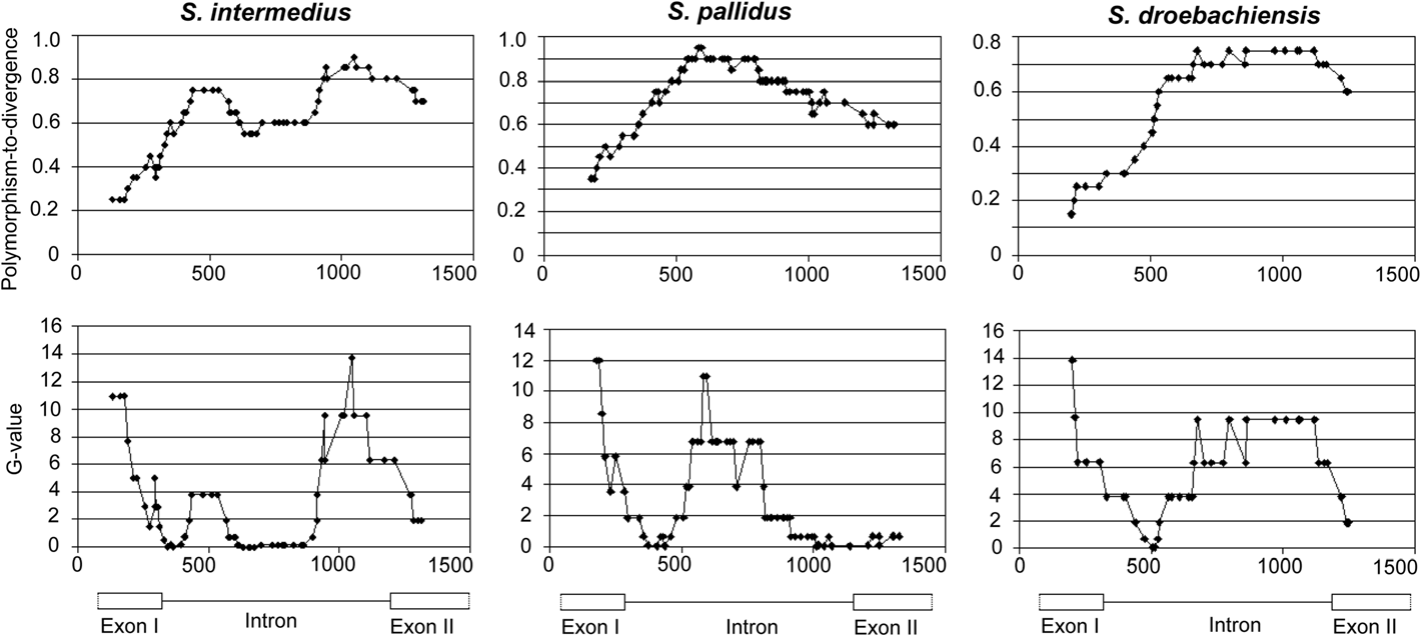
Sliding window plots of polymorphism-to-divergence ratio, and the average sliding G value along the *bindin* genes of *S. intermedius*, *S. pallidus*, and *S. droebachiensis*. The *bindin* sequence of *S. polyacanthus* (AF077317) was used as an outgroup. Window size is 10 variable substitutions for the polymorphism-to-divergence ratio and 12 variable substitutions for the average sliding G value. Other comments see Fig. 3

The McDonald tests [73, 74] revealed significant heterogeneity in the distribution of polymorphic sites along the *bindin* sequences (assessed by Monte Carlo simulations of the coalescent model incorporating recombination) and discordance between the levels of within species polymorphism and between species divergence (Table 2). Based on 10,000 simulations, with the recombination parameter varying from 1 to 64, the tests are significant for the *bindin* gene (Table 2). Two regions of the *bindin* gene have the largest average and maximum sliding G values (Fig. 4): (1) at the beginning of *bindin* exon I, which coincides with a region of low polymorphism-to-divergence ratio; and (2) in *bindin* intron, which coincides with the region of high polymorphism-to-divergence ratio (Figs. 3 and 4). The region of low polymorphism-to-divergence ratio is centered on exon I, with replacement substitutions in all three sea urchin species (Fig. 1, Additional file 2: Figures S2 and S3). The region of high polymorphism-to-divergence ratio is localized within the intron (Figs. 1, 3, and 4, Additional file 2: Figures S2 and S3). Low polymorphism-to-divergence ratio could result from directional selection, whereas high polymorphism-to-divergence ratio could result from balancing selection [73, 74]. Previously, we have shown that both types of selection are involved in the evolution of the *bap*, *lbe*, and *Est-6* genes of *D. melanogaster* [46, 47, 98, 99]. The present data suggest that both types of selection might be involved within the *bindin* gene of *Strongylocentrotus* species of sea urchins (below we show additional support for this possibility).

**Table 2:** McDonald tests

	G_max_		Runs		K.-S.		G_avg_	
	DRO	PAL	DRO	PAL	DRO	PAL	DRO	PAL
INT	22.220	17.264	34	36	0.090	0.062	5.666	6.306
*P* values	**0.012**	**0.059**	**0.081**	**0.059**	**0.035**	0.216	**0.031**	**0.019**
PAL	17.750	16.326	30	38	0.089	0.080	6.896	7.494
*P* values	**0.057**	**0.091**	**0.051**	0.491	**0.024**	**0.055**	**0.016**	**0.012**
DRO	--	27.985	--	25	--	0.116	--	9.278
*P* values	--	**0.002**	--	**0.038**	--	**0.011**	--	**0.002**

G_max_, Runs, Kolmogorov—Smirnov (K.-S.), and G_avg_ are test statistics (see [73, 74]). Marginally significant and significant *P* values are in bold. The *bindin* sequences of *S. droebachiensis* (AF133796) and *S. polyacanthus* (AF077317) were used in interspecific comparisons. Other comments see Table 1

The McDonald-Kreitman test [70] reveals significant deviation from neutrality for all three species, using coding and non-coding regions (Table 3): *S. intermedius* (G = 9.29, *P* = 0.0023), *S. pallidus* (G = 14.05, *P* = 0.0002), and *S. droebachiensis* (G = 5.68, *P* = 0.0172). However the test is not significant for any of the species if the noncoding region is excluded.

**Table 3:** McDonald-Kreitman test

	*S. intermedius*		*S. pallidus*		*S. droebachiensis*	
	Fixed	Polymorphic	Fixed	Polymorphic	Fixed	Polymorphic
Silent	24	45	17	59	20	33
Replacement	14	5	13	5	12	5
% Replacement	50.0	10.0	43.3	7.8	37.5	13.2
Fisher’s test	*P* = 0.0036		*P* = 0.0001		*P* = 0.0253	
G test	*P* = 0.0023		*P* = 0.0001		*P* = 0.0172	

The *bindin* sequence of *S. polyacanthus* (AF077317) was used for interspecific comparisons

With two sets of divergent haplotypes for the *bindin* gene of *S. intermedius* (Fig. 1), it is appropriate to use the haplotype test [72] to see whether directional selection has increased the frequency of some haplotypes. For the full dataset of 25 *bindin* sequences, there are a total of 30 parsimony informative polymorphic sites, and there is a homogeneous subset of 20 sequences with two informative sites (Fig 1). The probability of this configuration is significantly low (*P* = 0.03) with the population recombination rate 0.005 obtained by the method of McVean et al. [83]. The configuration is more asymmetric (the haplotype test *P* = 0.006) excluding recombinant sequence U-7 and sequence G-45 with two mutations in positions 219 and 244, Fig. 1). Thus, the homogeneous subset of sequences may evolve under the influence of directional selection. The region with amino acid substitutions (Fig. 1) at the beginning of *bindin* exon I may be a likely candidate as a target for directional selection (see also Fig. 4). The result of the haplotype test is consistent with the results of the McDonald tests (see above). For the other two species, *S. pallidus* and *S. droebachiensis*, the haplotype test is not significant, probably due to the very limited number of sequences available for these species.

**Fig. 5:**
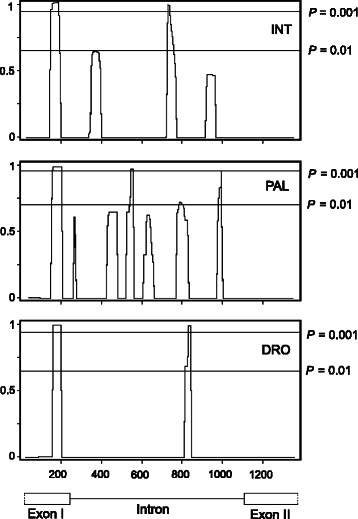
Sliding-window plots for the Wall’s (1999) *B* neutrality test statistic along the *bindin* gene region in *S. intermedius* (INT), *S. pallidus* (PAL), and *S. droebachiensis* (DRO). The total length of the sequence is 1398 bp with indels excluded. Window sizes are 50 nucleotides with five-nucleotide increments. Thin horizontal lines indicate expected values of the *B* neutrality test statistic with *P* = 0.01 (lower line) and *P* = 0.001 (upper line) obtained by coalescent simulations conditioned on the number of polymorphic sites without recombination. Other comments see Fig. 3

The neutrality tests of Hudson et al. [68], Tajima [69], Fu and Li [71], Depaulis and Veuille [76], Kelly [75], and Wall [77] are not significant. However, the sliding window analysis reveals a number of significant peaks of the neutrality tests based on linkage disequilibrium between segregating sites [75, 77]. Figure 5 illustrates the sliding-window plots for the Wall’s *B* neutrality test statistic [77]. The most pronounced peaks (*p*-values < 0.001 in coalescent simulations without recombination) are centered on the area of replacement polymorphism in exon I and the intron region (Fig. 5). Interestingly, the significant peaks frequently occur in the same coordinates (or very close) in all three sea urchins studied; moreover they coincide with the peaks of nucleotide variability (Fig. 3) and linkage disequilibrium (Additional file 4: Figure S4). Thus, the distribution of nucleotide variability is non-uniform and non-random along the *bindin* gene. Strong peaks of increased nucleotide variability accompanied by peaks of linkage disequilibrium and centered on the functionally important sites may reflect the effects of balancing selection, as it was predicted by theoretical analysis [65, 73–75, 77, 105, 107–110]. The patterns of polymorphism, divergence, and neutrality test statistics (Figs. 3, 4, and 5) suggest that the possible targets of selection in three congeneric sea urchins are localized in close sequence vicinities of the *bindin* gene, consistent with a parallel evolution in the *bindin* gene (see subsection “Sliding window analysis” above).

In general, the signal of positive selection could result from codon bias affecting fixed synonymous sites. The effective number of codons (ENC; [111]) ranges from 20, which means that the bias is at a maximum (so that only one codon is used from each synonymous codon group), to 61, which means no bias (all synonymous codons are equally used in each codon group). The values of ENC are 56.2, 52.1, and 57.1 for *S. intermedius*, *S. pallidus*, and *S. droebachinensis*, respectively; all numbers close to the maximum possible (61), indicating no codon bias in the *bindin* gene in the sea urchin studied.

The codon adaptation index (CAI; [112]) is a measure of the synonymous codon usage bias for a DNA or RNA sequence. The index ranges from 0 to 1, being 1 if a gene always uses the most frequently used synonymous codons in the reference set. We have calculated CAI using the original method proposed by Sharp and Li [112] implemented in the program E-CAI [113]. The random sequences are generated with the Markov method (Markov Model of order 0). The normality of the CAI values of the random generated sequences is assessed with a Kolmogorov-Smirnov test. The CAI values of the random-generated sequences are used to estimate an expected value of CAI that can be compared with the observed value. For the reference set of sequences we have used 1131 nucleotide sequences (410481 codons) of *S. purpuratus* from Codon Usage Database [114]. The observed CAI values are significantly lower than expected values (eCAI) (*P* = 0.01) for all three species studied, indicating no codon usage bias in the *bindin* gene. For *S. intermedius* CAI = 0.643, eCAI = 0.734; *P* = 0.01; for *S. pallidus* CAI = 0.668, eCAI = 0.750; *P* = 0.01, and for *S. droebachiensis* CAI = 0.663, eCAI = 0.754; *P* = 0.01.

The maximum likelihood analysis of positive selection (see below) cannot be biased due to the effects of selection for codon bias since unequal codon frequencies are explicitly taken into account within the codon model. Thus the positive selection is detected given the background codon bias.

The neutrality tests are typically affected by demography and, therefore, they may be difficult to interpret [115, 116]. We have applied model-based maximum likelihood (ML) methods to confirm the observations made above. All results from the ML analyses shown below held for the full sample of *S. intermedius*, *S. pallidus*, and *S. droebachiensis*, whether or not recombinant sequences were removed.

### Maximum likelihood analysis of the coding regions

Three LRTs (M0 vs. M3, M1a vs. M2a, and M7 vs M8) were applied to test for positive selection on the protein in separate alignments of *S. intermedius*, *S. pallidus*, *S. droebachiensis* and the alignment of several other sea urchin species. None of these tests is significant for the *S. intermedius* and *S. droebachiensis* alignments. Model M0 offered equally good fit to data as more flexible models. It was estimated that the *bindin* gene evolved with *ω* = 0.7 and *κ* = 4.3 in *S. intermedius* and with *ω* = 0.5 and *κ* = 2.1 in *S. droebachiensis*. However, it seems unlikely that all sites in the gene are under the same selective pressure, due to constraints imposed by tertiary protein structure and function. We attribute such a result to the low power of LRTs for datasets of low divergence [117]. For the *S. pallidus* alignment, all three LRT are significant with *p*-values from 0.01 to 0.02. The ML estimates suggest that 97 % of sites in *bindin* are very conserved, but the remaining 3 % of sites evolve under strong diversifying selective pressure with ω = 37.3.

For the species alignment, all three tests are highly significant (*p*-values ≤ 0.0001). ML parameter estimates suggest that about 75 % of sites evolve under variable pressures of purifying selection (from strict to relaxed); 20 % of sites evolve nearly-neutrally; and 4 % of sites evolve under positive selection pressure with ω_2_ = 9.56. Sites under positive selection predicted by a Bayesian approach are listed in Table 4.

**Table 4:** Sites inferred under positive selection using the Bayesian prediction based on codon models M2a and M8

Data set	Positively selected sites	M2a NEB	M2a BEB	M8 NEB	M8 BEB
All species	2 P/V/G	0.941	0.945	0.962*	0.974*
	35 G/R/A	0.825	0.842	0.862	0.901
	74 I/F/V/I/T	0.642	0.704	0.704	0.793
	160 F/L	0.745	0.747	0.763	0.801
	188 V/G/S/A	1.000**	1.000**	1.000**	1.000**
	199 L/Q/R	0.993**	0.991*	0.996**	0.996**
*S. pallidus*	66 I/L	1.000**	0.668	1.000**	0.777
	162 P/V	1.000**	0.816	1.000**	0.904
	188 A/G	1.000**	NA	1.000**	0.580
	199 L/I	1.000**	NA	1.000**	0.582

Site numbers are mapped to the full species alignment after the intron sites were removed

*: *P* > 95 %; **: *P* > 99 %. See Additional file 5: Figure S5 for the bindin amino acid alignment

The site models rely on the unique inferred phylogeny. For population data, phylogenetic inference may lack resolution due to low divergence or may be inaccurate due to recombination. Although site models are rather robust to recent deviations in topological arrangement, it is advantageous to use other techniques with different assumptions to check the robustness of the conclusions, and to see whether additional conclusions can be drawn from different types of analyses.

Therefore, in addition we used the PAC likelihood method based on the approximation to the coalescent with selection and recombination parameters estimated simultaneously in the Bayesian framework [118]. The method was applied to the three population samples above (*S. intermedius*, *S. pallidus*, and *S. droebachiensis*). Sites under positive selection can be inferred on a site-to-site basis or in sliding windows, and the posterior probabilities at each site can be used to measure the confidence of the prediction. However, unlike LRT the technique does not intend to test the hypothesis of positive selection on the gene overall. In this framework, this would be equivalent to answering the question, “Is there at least one site under positive selection in the gene?” Posed this way, the problem can be seen as similar to multiple testing, whereby the hypothesis of positive selection is tested at each site using posterior probabilities rather than *p*-values as is usually done (and it is not meant to complement the Bayesian framework). However, for explorative purposes, we apply the Benjamini and Hochberg [119] multiple-testing correction at each site (1- posterior probability) instead of a *p*-value.

In all three species alignments, several sites were inferred to be under positive selection with posterior probability > 0.95. When sequences for the U and G forms of *S. intermedius* were analyzed separately, more sites under positive selection were inferred for the U form (excluding U7) compared to the G form, which is consistent with the observation of higher variability in the U form (above). When all *bindin* sequences of *S. intermedius* were analyzed together, 10 sites were inferred under positive selection. After the multiple testing correction, some sites were still under positive selection in each of the datasets (according to the full species alignment: sites 65, 67, 87, 160, 163 and 185 in *S. droebachiensis*; sites 65 and 199 in *S. intermedius*; and sites 188 and 199 in *S. pallidus*; see Additional file 5: Figure S5 for the bindin amino acid alignment). These results suggest that diversifying selection acts both at the species and population level of the sea urchins studied. Similar results were obtained previously for the *bindin* coding region of the sea urchins. A number of sites evolving under positive selection were detected in the 5′ and 3′ bindin regions of the sea urchins genera *Strongylocentrotus* [25, 26] and *Echinomenta* [17, 22, 23]. Our results are completely compatible with the previous studies. We found three sites under positive selection both in the 5′ and 3′ regions, but none within the core.

### Testing for positive selection in the intron region

To test for evidence of positive selection in the *bindin* intron, we used the ML method of Wong and Nielsen [92], which compares the rate of nucleotide change in the intron to the rate of synonymous changes in the coding region. This ratio is denoted as ζ and can vary among intron sites. Sites with ζ > 1 are under positive selection acting on the non-coding region. Two nested models were fitted to data: a neutral model that does not allow ζ > 1 and a two-category model that allows ζ > 1 (see Table 5). The double likelihood difference is then compared to the 50:50 mixture of *χ*_0_^2^ and *χ*_1_^2^, to test whether the two-category model fits data significantly better.

**Table 5:** Models of variable ζ among sites

Model	Free parameters	Site classes	Proportions of sites from a corresponding class
Neutral	ζ_0,_ *p*_0_	ζ_0_ < 1, ζ_1_ = 1	*p*_0,_ *p*_1_ = 1− *p*_0_
Two-category	ζ_0,_ ζ_1,_ *p*_0_	ζ_0_ < 1, ζ_1_ ≥ 1	*p*_0,_ *p*_1_ = 1− *p*_0_

LRTs for positive selection on the noncoding region were significant in all three datasets (see Table 6 for estimates and *p*-values); the estimated proportions of intron sites evolving under positive selection were 2 % in *S. intermedius* and 9 % in *S. droebachiensis*. In *S. pallidus* the whole intron was suggested to be under positive diversifying selection, which may act homogeneously on intron sites, since we could not reject the homogeneity of evolutionary rates in this intron (unlike for *S. intermedius* and *S. droebachiensis*). To be sure that this result is not an estimation problem, we tested the hypothesis of homogeneous rate for all sites in the intron region using baseml from PAML. For *S. intermedius* and *S. droebachiensis* the hypothesis was rejected – rates are non-homogeneous, consistent with Table 6; but for *S. pallidus* we could not reject homogeneity. Consequently, the entire *bindin* intron might constitute a target of positive selection in *S. pallidus*.

**Table 6:** Parameter estimates for ζ-models

Data	Model	κ	ω	ζ-model parameters^a^	Log-likelihood values
INT	Neutral	3.30	0.49	ζ_0_ = 0.99, *p*_0_ = 0.14 [ζ_1_ = 1, *p*_1_ = 0.86]	−2886.217945
	Two-category	2.62	0.53	ζ_0_ = 0.69, *p*_0_ = 0.98 ζ_1_ = 61.77, [*p*_1_ = 0.02]	−2870.740681 *p*-value = 10^−8^
PAL	Neutral	3.12	0.27	ζ_0_ = 1.00, *p*_0_ = 0.00 [ζ_1_ = 1, *p*_1_ = 1.00]	−2533.273804
	Two-category	2.87	2.12	ζ_0_ = 0.00, *p*_0_ = 0.00 ζ_1_ = 8.80, [*p*_1_ = 1.00]	−2522.600909 *p*-value = 2x10^−6^
DRO	Neutral	1.14	0.31	ζ_0_ = 0.00, *p*_0_ = 0.53 [ζ_1_ = 1, *p*_1_ = 0.47]	−2279.474923
	Two-category	1.17	0.41	ζ_0_ = 0.00, *p*_0_ = 0.91 ζ_1_ = 7.27, [*p*_1_ = 0.09]	−2277.438710 *p*-value = 0.02

^a^Values in square brackets are fixed (ζ_1_ in neutral model) or calculated from estimates (*p*_1_ = 1− *p*_0_)

See Table 1 for the species designation

A number of evolutionary processes in the *bindin* coding region such as mildly deleterious selection against unpreferred synonymous codons, selective sweeps due to positive selection on nonsynonymous variation, and background selection could potentially influence the results we detected in the *bindin* intron. However, these possible alternative mechanisms represent the variants of selection that decrease variability (e.g., [120–122]). Contrary to these scenarios we observe highly increased level of variability in the intron, moreover accompanied by a decreased level of divergence, indicating the action of diversifying selection [65, 73–75, 77, 105, 107–110] operating within the *bindin* intron independently from the coding region.

We report several tests that support positive selection on the *bindin* intron. The statistical significance of the deviation from neutrality in the intron region is supported not only by the ML analysis based on joint codon-nucleotide models but also by several neutrality tests - Kelly [75], Wall [77], and McDonald [73, 74]. The observed consistency between different approaches is an important argument supporting the results obtained in this work.

Further, the results of the ML analysis are likely to be robust. First, the selection measure relies on the *d*_*S*_ measured as average over the entire coding region. Since there is not even weak codon bias in the coding region (according to codon bias indices, see above), average *d*_*S*_ cannot be affected to inflate the measure of selection as a whole. Site-specific synonymous bias events, if present, are insufficient to affect the average *d*_*S*_ significantly. Therefore there is nothing undermining the ML analysis of selection on the *bindin* intron of the strongylocentrotid sea urchins.

The data obtained here are in accordance with other investigations where positive selection acting at the intron sites was revealed or suggested: in the plant *Ficus carica* [123], *Drosophila melanogaster* [46, 47, 124], and primates including humans [125–129]. Nevertheless, the results of the neutrality tests and the interaction between selective and neutral processes should be cautiously interpreted, given the modest sample size of sequences with the relatively short sequence lengths from a single population (e.g., [130, 131]). Moreover, there are nonselective factors that could partly account for the patterns of the *bindin* polymorphisms. Possible explanatory processes include bottlenecks and founding effects and/or population (or species) admixture, as well as varying recombination rates in different genomic regions.

Demographic and selective forces shaping nucleotide polymorphism patterns in strongylocentrotid sea urchin species are difficult to disentangle because of their highly complicated evolutionary history, including wide dispersal in the North Atlantic, Arctic, and Pacific continental shelves and slopes, and adaptation to drastically new environments [49, 50]. Previously we showed that the patterns of polymorphism should be influenced by both of these evolutionary forces and is apparent in our data obtained for the *Sod*, *Est-6*, ψ*Est-6*, *tin*, *bap*, *lbe*, and *lbl* genes from four natural *Drosophila melanogaster* populations (Africa, Europe, North and South America) [46, 47, 97–99]. Comparative analysis showed significant peaks of variability observed both in African and non-African samples, but dimorphic structure was detected only in non-African samples. This observation supports the hypothesis that dimorphic haplotype structure could be generated by demographic process during the recent species history caused by admixture of differentiated populations (or species). Indeed, deep branches due to demographic factors are expected in coalescent theory [132]. Moreover, there is direct evidence for introgression between *S. pallidus* and *S. droebachiensis* [44, 55]. However, the elevated levels of nucleotide variability and linkage disequilibrium, accompanied by significant peaks of neutrality test statistics could not be explained by stochastic processes alone and might reflect the effects of balancing selection [65, 73–75, 77, 105, 107–110]. One way of distinguishing between selective and demographic processes could be to perform similar investigations in other populations of strongylocentrotid sea urchins combined with a functional approach using experimental methods to correlate specific *bindin* haplotypes with specific functional differences related to reproduction. This integral approach promises to be fruitful, thus adding a new aspect in evolutionary studies of *bindin* including the intron sequences mostly overlooked in previous investigations.

## Conclusions

1. We have studied nucleotide variability in the complete *bindin* locus including two exons and one intron in the sea urchin *S. intermedius* (including two morphological forms) and in two other strongylocentrotid species, *S. pallidus* and *S. droebachiensis* available in GenBank. The *bindin* gene introns have not been previously investigated for any species of sea urchins.
2. The distribution of variability and divergence is non-uniform along *bindin*, with striking similarity between all three species, indicating similar evolutionary trends of this gene with a reproductive function. The revealed pattern is consistent with the phenomenon of parallel evolution, which results from similar or identical mutations maximizing adaptation in independent evolutionary lineages. This suggestion is supported by significant interspecific correlations of nucleotide diversity patterns from sliding windows over the *bindin* gene.
3. The patterns of nucleotide variability and divergence are radically different in the *bindin* coding and intron regions. The signature of positive selection is detected in the *bindin* coding region. Moreover, the data suggest the action of diversifying selection in the *bindin* intron, which to our knowledge has not been shown previously. Different types of positive selection are suggested in different functional regions, with putative multiple targets of selection both in coding and intron regions. Significant deviation from neutrality suggests functional importance of the *bindin* intron variability, which might be involved in regulatory functions.
4. The morphological forms of *S. intermedius* show no evidence of genetic divergence. However they demonstrate slightly different patterns of *bindin* variability. These observations along with clear morphological and ecological differences, as well as the highly specific symbiotic microorganisms previously found [54], suggest unique evolutionary trajectories for each form and warrant treating them separately with respect to biodiversity conservation and management.

### Ethics (and consent to participate)

The sea urchin *Strongylocentrotus intermedius* is not listed as endangered, vulnerable, rare, or protected species of the Russian Federation. The *S. intermedius* is considered as a “commercial” species in the Primorye Territory (the Sea of Japan) of the Russian Federation, where we collected the specimens for the present study. Fishery of *S. intermedius* is officially permitted and administered.

The described field study was based on the quota limit obtained from the Department of Fisheries and Marine Resources of Primorye Territory (DFMRPT) (order #152, December 12, 2014; signed by the DFMRPT Director A.A. Perednya; see details, http://primorsky.ru/upload/iblock/dee/deed8734a1207787f7658b55df06b654.pdf). The sampling point is located beyond any protected territories. The field study did not involve endangered, vulnerable, rare, or protected species. The locations of the field studies are not privately-owned or protected.

The present field study was approved by the Federal Agency for Fishery of the Russian Federation, which has the highest decision authority concerning marine organisms care and use and should be considered as an equivalent to the Institutional Animal Care and Use Committee.

The funders had no role in study design, data collection and analysis, decision to publish, or preparation of the manuscript. The authors alone are responsible for the content and writing of the paper.

### Consent to publish

Not applicable.

### Availability of data and materials

The nucleotide sequences obtained in the present work are deposited in GenBank (National Center for Biotechnology Information) under the accession numbers: KP774723—KP774781 (*COI*) and KP774782—KP774794 (*bindin*) at www.ncbi.nlm.nih.gov [133].

## Additional files

Additional file 1:
**Text S1.** PCR reactions. **Text S2.** GenBank *bindin* sequences. (DOC 68 kb) Additional file 2: Figure S1. DNA polymorphism in the *bindin* repeat region (exon II) of *Strongylocentrotus intermedius*. **Figure S2.** DNA polymorphism in the *bindin* gene of *Strongylocentrotus pallidus*. The recombinant sequences (PAL_AF133806 and PAL_AF077313) are in bold. The exon—intron coordinates are: 1–237: exon I; 238–1189: intron; 1190–1543: exon II. Other comments see Fig. 1. **Figure S3.** DNA polymorphism in the *bindin* gene of *Strongylocentrotus droebachiensis*. The long 535-bp insertion has been deleted (see text for details). The exon - intron coordinates are: 1–237: exon I; 238–1189: intron; 1190–1516: exon II. Other comments see Fig. 1. **Text S3.** Repeat region. **Text S4.** Indel coordinates. (DOC 75 kb) Additional file 3: Table S1. Recombination estimates with RDP3. **Table S2.** The *bindin* gene recombination estimates (*ρ*). (DOC 45 kb) Additional file 4: Figure S4. Sliding window plots of linkage disequilibrium (measured by *D*) along the *bindin* gene region in *S. intermedius* (INT), *S. pallidus* (PAL), and *S. droebachiensis* (DRO). Indels are excluded (left side) or included (right side). Window sizes are 60 nucleotides with 25-nucleotide increments. A schematic representation of the *bindin* gene is displayed at the bottom. (TIF 584 kb) Additional file 5: Figure S5. Bindin amino acid alignment. INT = *S. intermedius*, DRO = *S. droebachiensis*, PAL = *S. pallidus*; POL = *S. polyacanthus*; PUR = *S. purpuratus*; PUL = *Hemicentrotus pulcherrimus*. (PNG 178 kb)

## References

[1] Vacquier VD, Swanson WJ, Hellberg ME (1995). What have we learned about sea urchin sperm bindin?. Dev Growth Differ.

[2] Swanson WJ, Vacquier VD (2002). Reproductive protein evolution. Annu Rev Ecol Syst.

[3] Zigler KS, Lessios HA (2003). 250 million years of bindin evolution. Biol Bull.

[4] Zigler KS (2008). The evolution of sea urchin sperm bindin. Int J Dev Biol.

[5] Palumbi SR (2009). Speciation and the evolution of gamete recognition genes: patterns and process. Heredity.

[6] Lessios HA (2011). Speciation genes in free-spawning marine invertebrates. Integrat Compar Biol.

[7] Vacquier VD, Swanson WJ (2011). Selection in the rapid evolution of gamete recognition proteins in marine invertebrates. Cold Spring Harb Perspect Biol.

[8] Vacquier VD (2012). The quest for the sea urchin egg receptor for sperm. Biochem Biophys Res Com.

[9] Cameron RA, Minor JE, Nishioka D, Britten RJ, Davidson EH (1990). Locate and level of bindin mRNA in maturing testis of the sea urchin, *Strongylocentrotus purpuratus*. Dev Biol.

[10] Nishioka D, Ward RD, Poccia D, Kostacos C, Minor JE (1990). Localization of bindin expression during sea urchin spermatogenesis. Mol Reprod Dev.

[11] Summers RG, Hylander BL, Colwin LH, Colwin AL (1975). The functional anatomy of the echinoderm spermatozoon and its interaction with the egg at fertilization. Am Zool.

[12] Metz EC, Kane RE, Yanagimachi H, Palumbi SR (1994). Fertilization between closely related sea urchins is blocked by incompatibilities during sperm-egg attachment and early stages of fusion. Biol Bull.

[13] Swanson WJ, Vacquier VD (2002). The rapid evolution of reproductive proteins. Nat Rev Genet.

[14] Palumbi SR (1999). All males are not created equal: fertility differences depend on gamete recognition polymorphisms in sea urchins. Proc Natl Acad Sci U S A.

[15] Levitan DR, Ferrell DL (2006). Selection on gamete recognition proteins depends on sex, density, and genotype frequency. Science.

[16] Levitan DR, Stapper AP (2009). Simultaneous positive and negative frequency-dependent selection on sperm bindin, a gamete recognition protein in the sea urchin *Strongylocentrotus purpuratus*. Evolution.

[17] Zigler KS, McCartney MA, Levitan DR, Lessios HA (2005). Sea urchin bindin divergence predicts gamete compatibility. Evolution.

[18] Geyer LB, Palumbi SP (2003). Reproductive character displacement and the genetics of gamete recognition in tropical sea urchins. Evolution.

[19] Ulrich AS, Otter M, Glabe CG, Hoekstra D (1998). Membrane fusion is induced by a distinct peptide sequence of the sea urchin fertilization protein bindin. J Biol Chem.

[20] Patiño S, Aagaard JE, Maccoss MJ, Swanson WJ, Hart MW (2009). Bindin from a sea star. Evol Dev.

[21] Lopez A, Miraglia SJ, Glabe CG (1993). Structure/function analysis of the sea urchin sperm adhesive protein bindin. Dev Biol.

[22] Metz EC, Palumbi SR (1996). Positive selection and sequence rearrangements generate extensive polymorphism in the gamete recognition protein bindin. Mol Biol Evol.

[23] McCartney MA, Lessios HA (2004). Adaptive evolution of sperm bindin tracks egg incompatibility in neotropical sea urchins of the genus *Echinometra*. Mol Biol Evol.

[24] Geyer LB, Lessios H (2009). Lack of character displacement in the male recognition molecule, bindin, in Atlantic sea urchins of the genus *Echinometra*. Mol Biol Evol.

[25] Biermann CH (1998). The molecular evolution of sperm bindin in six species of sea urchins (Echinoida: Strongylocentrotidae). Mol Biol Evol.

[26] Pujolar JM, Pogson GH (2011). Positive Darwinian selection in gamete recognition proteins of *Strongylocentrotus* sea urchins. Mol Ecol.

[27] Zigler KS, Raff EC, Popodi E, Raff RA, Lessios HA (2003). Adaptive evolution of bindin in the genus *Heliocidaris* is correlated with the shift to direct development. Evolution.

[28] Calderón I, Turon X, Lessios HA (2009). Characterization of the sperm molecule bindin in the sea urchin genus *Paracentrotus*. J Mol Evol.

[29] Calderón I, Ventura CRR, Turon X, Lessios HA (2010). Genetic divergence and assortative mating between colour morphs of the sea urchin *Paracentrotus gaimardi*. Mol Ecol.

[30] Metz EC, Gomes-Gutierez G, Vacquier VD (1998). Mitochondrial DNA and bindin gene sequence evolution among allopatric species of the sea urchin genus *Arbacia*. Mol Biol Evol.

[31] Lessios HA, Lockhart S, Collin R, Sotil G, Sanchez-Jerez P, Zigler KS (2012). Phylogeography and bindin evolution in *Arbacia*, a sea urchin genus with an unusual distribution. Mol Ecol.

[32] Zigler KS, Lessios HA (2003). Evolution of bindin in the pantropical sea urchin *Tripneuster*: comparisons to bindin of other genera. Mol Biol Evol.

[33] Zigler KS, Lessios HA (2004). Speciation on the coasts of the new world: phylogeography and the evolution of bindin in the sea urchin genus *Lytechinus*. Evolution.

[34] Debenham P, Brzezinski MA, Foltz KR (2000). Evaluation of sequence variation and selection in the bindin locus of the red sea urchin, *Strongylocentrotus franciscanus*. J Mol Evol.

[35] Sunday JM, Hart MW (2013). Sea star populations diverge by positive selection at a sperm-egg compatibility locus. Evol Ecol.

[36] Popovic I, Marko PB, Wares JP, Hart MW (2014). Selection and demographic history shape the molecular evolution of the gamete compatibility protein bindin in *Pisaster* sea stars. Ecol Evol.

[37] Dobzhansky T (1940). Speciation as a stage in evolutionary divergence. Am Nat.

[38] Eberhard WG (1996). Female Control: Sexual Selection by Cryptic Female Choice.

[39] Rice WR, Holland B (1997). The enemies within: Intergenomic conflict. Interlocus contest evolution, ICE and the intraspecific red queen. Behav Ecol Sociobiol.

[40] Gould MC, Stephano JL (2003). Polyspermy prevention in marine invertebrates. Microscopy Research and Technology.

[41] Vacquier VD, Swanson WJ, Lee YH (1997). Positive Darwinian selection on two homologous fertilization proteins: what is the selective pressure driving their divergence?. J Mol Evol.

[42] Zigler KS, Byrne M, Raff EC, Lessios HA, Raff RA (2012). Natural hybridization in the sea urchin genus *Pseudoboletia* between species without apparent barriers to gamete recognition. Evolution.

[43] Binks RM, Prince J, Evans JP, Kennington WJ (2012). More than bindin: reproductive isolation between sympatric subspecies of a sea urchin by asynchronous spawning. Evolution.

[44] Addison JA, Pogson GH (2009). Multiple gene genealogies reveal asymmetrical hybridization and introgression among strongylocentrotid sea urchins. Mol Ecol.

[45] Chorev M, Carmel L (2012). The function of introns. Front Genet.

[46] Balakirev ES, Ayala FJ (2004). Nucleotide variation in the *tinman* and *bagpipe* homeobox genes of *Drosophila melanogaster*. Genetics.

[47] Balakirev ES, Anisimova M, Ayala FJ (2011). Complex interplay of evolutionary forces in the *ladybird* homeobox genes of *Drosophila melanogaster*. PLoS One.

[48] Kober KM, Bernardi G (2013). Phylogenomics of strongylocentrotid sea urchins. BMC Evol Biol.

[49] Jensen M (1974). The Strongylocentrotidae (Echinoidea), a morphologic and systematic study. Sarsia.

[50] Bazhin AG, Stepanov VG (2012). Sea urchins fam. Strongylocentrotidae of seas of Russia.

[51] Swan EF (1962). Evidence suggesting the existence of two species of *Strongylocentrotus* (Echinoidea) in the northwest Atlantic. Can J Zool.

[52] Gagnon J-M, Gilkinson KD (1994). Discrimination and distribution of the sea urchins *Strongylocentrotus droebachiensis* (O.F. Müller) and *S. pallidus* (G.O. Sars) in the north-west Atlantic. Sarsia.

[53] Buyanovsky AI, Rzhavsky AV (2007). Spatial structure of settlements of green sea urchin *Strongylocentrotus droebachiensis* (Echinodermata; Strongylocentrotidae) in the Dalne-Zelenetskaya inlet in the Barents sea. Proceedings of the Russian Federal Research Institute of Fisheries and Oceanography.

[54] Balakirev ES, Pavlyuchkov VA, Ayala FJ (2008). DNA variation and symbiotic associations in phenotypically-diverse sea urchin *Strongylocentrotus intermedius*. Proc Natl Acad Sci U S A.

[55] Addison JA, Hart MW (2005). Colonization, dispersal, and hybridization influence phylogeography of North Atlantic sea urchins (*Strongylocentrotus droebachiensis*). Evolution.

[56] Balakirev ES, Krupnova TN, Ayala FJ (2012). Symbiotic associations in the phenotypically-diverse brown alga *Saccharina japonica*. PLoS One.

[57] Balakirev ES, Romanov NS, Mikheev PB, Ayala FJ (2013). Mitochondrial DNA variation and introgression in Siberian taimen *Hucho taimen*. PLoS One.

[58] Minor JE, Fromson DR, Britten RJ, Davidson EH (1991). Comparison of the bindin proteins of *Strongylocentrotus franciscanus*, *S. purpuratus*, and *Lytechinus variegatus*: sequences involved in the species specificity of fertilization. Mol Biol Evol.

[59] Gao B, Klein LE, Britten RJ, Davidson EH (1986). Sequence of mRNA coding for bindin, a species-specific sea urchin sperm protein required for fertilization. Proc Natl Acad Sci U S A.

[60] Lee YH (2003). Molecular phylogenies and divergence times of sea urchin species of strongylocentrotidae, echinoida. Mol Biol Evol.

[61] Jacobs HT, Elliott DJ, Math VB, Farquharson A (1988). Nucleotide sequence and gene organization of sea urchin mitochondrial DNA. J Mol Biol.

[62] Thompson JD, Higgins DG, Gibson TJ (1994). CLUSTAL W: improving the sensitivity of progressive multiple sequence alignment through sequence weighting, position-specific gap penalties and weight matrix choice. Nucleic Acids Res.

[63] Librado P, Rozas J (2009). DnaSP v5: A software for comprehensive analysis of DNA polymorphism data. Bioinformatics.

[64] Filatov DA (2002). PROSEQ: a software for preparation and evolutionary analysis of DNA sequence data sets. Mol Ecol Notes.

[65] Hudson RR, Kaplan N (1988). The coalescent process in models with selection and recombination. Genetics.

[66] Tamura K, Peterson D, Peterson N, Stecher G, Nei M, Kumar S (2011). MEGA5: Molecular evolutionary genetics analysis using maximum likelihood, evolutionary distance, and maximum parsimony methods. Mol Biol Evol.

[67] Balakirev ES, Krupnova TN, Ayala FJ. DNA variation in the phenotypically-diverse brown alga Saccharina japonica. BMC Plant Biol. 2012;12(108). doi:10.1186/1471-2229-12-108. 10.1186/1471-2229-12-108 PMC3490969 22784095

[68] Hudson RR, Kreitman M, Aguadé M (1987). A test of neutral molecular evolution based on nucleotide data. Genetics.

[69] Tajima F (1989). Statistical method for testing the neutral mutation hypothesis by DNA polymorphism. Genetics.

[70] McDonald JH, Kreitman M (1991). Adaptive protein evolution at the *Adh* locus in Drosophila. Nature.

[71] Fu Y-X, Li W-H (1993). Statistical tests of neutrality of mutations. Genetics.

[72] Hudson RR, Bailey K, Skarecky D, Kwiatowski J, Ayala FJ (1994). Evidence for positive selection in the superoxide dismutase (*Sod*) region of *Drosophila melanogaster*. Genetics.

[73] McDonald JH (1996). Detecting non-neutral heterogeneity across a region of DNA sequence in the ratio of polymorphism to divergence. Mol Biol Evol.

[74] McDonald JH (1998). Improved tests for heterogeneity across a region of DNA sequence in the ratio of polymorphism to divergence. Mol Biol Evol.

[75] Kelly JK (1997). A test of neutrality based on interlocus associations. Genetics.

[76] Depaulis F, Veuille M (1998). Neutrality tests based on the distribution of haplotypes under an infinite-site model. Mol Biol Evol.

[77] Wall JD (1999). Recombination and the power of statistical tests of neutrality. Genet Res.

[78] Hudson RR, Boos D, Kaplan NL (1992). A statistical test for detecting geographic subdivision. Mol Biol Evol.

[79] Hudson RR (1983). Properties of a neutral allele model with intragenic recombination. Theor Popul Biol.

[80] Hudson RR (1990). Gene genealogies and the coalescent process. Oxf Surv Biol.

[81] Hudson RR (2002). Generating samples under a Wright-Fisher neutral model of genetic variation. Bioinformatics.

[82] Sawyer SA (1989). Statistical tests for detecting gene conversion. Mol Biol Evol.

[83] McVean G, Awadalla P, Fearnhead P (2002). A coalescent-based method for detecting and estimating recombination from gene sequences. Genetics.

[84] Martin DP, Lemey P, Lott M, Moulton V, Posada D, Lefeuvre P (2010). RDP3: a flexible and fast computer program for analyzing recombination. Bioinformatics.

[85] Guindon S, Dufayard JF, Lefort V, Anisimova M, Hordijk W, Gascuel O (2010). New algorithms and methods to estimate maximum-likelihood phylogenies: assessing the performance of PhyML 3.0. Syst Biol.

[86] Yang Z, Swanson WJ, Vacquier VD (2000). Maximum-likelihood analysis of molecular adaptation in abalone sperm lysin reveals variable selective pressures among lineages and sites. Mol Biol Evol.

[87] Yang Z, Nielsen R (2008). Mutation-selection models of codon substitution and their use to estimate selective strengths on codon usage. Mol Biol Evol.

[88] Yang Z (2007). PAML 4: phylogenetic analysis by maximum likelihood. Mol Biol Evol.

[89] Kosakovsky Pond SL, Muse SV (2005). Site-to-site variation of synonymous substitution rates. Mol Biol Evol.

[90] Kosakovsky Pond SL, Frost SDW, Muse SV (2005). HyPhy: hypothesis testing using phylogenies. Bioinformatics.

[91] Anisimova M, Kosiol C (2009). Investigating protein-coding sequence evolution with probabilistic codon substitution models. Mol Biol Evol.

[92] Wong WS, Nielsen R (2004). Detecting selection in noncoding regions of nucleotide sequences. Genetics.

[93] Yang Z, Wong WS, Nielsen R (2005). Bayes empirical bayes inference of amino acid sites under positive selection. Mol Biol Evol.

[94] Moy GW, Vacquier VD (2008). Bindin genes of the Pacific oyster *Crassostrea gigas*. Gene.

[95] Du J, Gu T, Tian H, Araki H, Yang Y-H, Tian D (2008). Grouped nucleotide polymorphism: a major contributor to genetic variation in *Arabidopsis*. Gene.

[96] Teeter K, Naeemuddin M, Gasperini R, Zimmerman E, White KP, Hoskins R (2000). Haplotype dimorphism in a SNP collection from *Drosophila melanogaster*. J Exp Zool.

[97] Balakirev ES, Chechetkin VR, Lobzin VV, Ayala FJ (2003). DNA polymorphism in the β-esterase gene cluster of *Drosophila melanogaster*. Genetics.

[98] Balakirev ES, Ayala FJ (2003). Nucleotide variation of the *Est-6* gene region in natural populations of *Drosophila melanogaster*. Genetics.

[99] Balakirev ES, Balakirev EI, Ayala FJ (2002). Molecular evolution of the *Est-6* gene in *Drosophila melanogaster*: Contrasting patterns of DNA variability in adjacent functional regions. Gene.

[100] Hudson RR, Sáez AG, Ayala FJ (1997). DNA variation at the *Sod* locus of *Drosophila melanogaster*: an unfolding story of natural selection. Proc Natl Acad Sci U S A.

[101] Aguadé M (2001). Nucleotide sequence variation at two genes of the phenylpropanoid pathway, the *FAH1* and *F3H* genes, in *Arabidopsis thaliana*. Mol Biol Evol.

[102] Haubold B, Kroymann J, Ratzka A, Mitchell-Olds T, Wiehe T (2002). Recombination and gene conversion in a 170-kb genomic region of *Arabidopsis thaliana*. Genetics.

[103] Hudson RR, Kaplan N (1985). Statistical properties of the number of recombination events in the history of a sample of DNA sequences. Genetics.

[104] Moy GW, Springer SA, Adams SL, Swanson WJ, Vacquier VD (2008). Extraordinary intraspecific diversity in oyster sperm bindin. Proc Natl Acad Sci U S A.

[105] Nordborg M (1997). Structured coalescent processes on different time scales. Genetics.

[106] Stern DL (2013). The genetic causes of convergent evolution. Nature Rev Genet.

[107] Strobeck C (1983). Expected linkage disequilibrium for a neutral locus linked to a chromosomal arrangement. Genetics.

[108] Charlesworth B, Nordborg M, Charlesworth D (1997). The effects of local selection, balanced polymorphism and background selection on equilibrium patterns of genetic diversity in subdivided populations. Genet Res.

[109] Takahata N, Satta Y (1998). Footprints of intragenic recombination at HLA loci. Immunogenetics.

[110] Charlesworth D (2006). Balancing selection and its effects on sequences in nearby genome regions. PLoS Genet.

[111] Wright F (1990). The “effective number of codons” used in a gene. Gene.

[112] Sharp PM, Li WH (1987). The codon adaptation index – a measure of directional synonymous codon usage bias, and its potential applications. Nucleic Acids Res.

[113] Puigbò P, Bravo IG, Santiago G-VS (2008). CAIcal: A combined set of tools to assess codon usage adaptation. Biol Direct.

[114] Nakamura Y, Gojobori T, Ikemura T (2000). Codon usage tabulated from international DNA sequence databases: status for the year 2000. Nucleic Acids Res.

[115] Wayne ML, Simonsen K (1998). Statistical tests of neutrality in the age of weak selection. Trends Ecol Evol.

[116] Nielsen R (2001). Statistical tests of selective neutrality in the age of genomics. Heredity.

[117] Anisimova M, Bielawski JP, Yang Z (2001). Accuracy and power of the likelihood ratio test in detecting adaptive molecular evolution. Mol Biol Evol.

[118] Wilson DJ, Mcvean G (2006). Estimating diversifying selection and functional constraint in the presence of recombination. Genetics.

[119] Benjamini Y, Hochberg Y (1995). Controlling the false discovery rate: a practical and powerful approach to multiple testing. J Royal Statist Soc Ser B.

[120] Smith JM, Haigh J (1974). The hitch-hiking effect of a favourable gene. Genet Res.

[121] Charlesworth B, Morgan MT, Charlesworth D (1993). The effect of deleterious mutations on neutral molecular variation. Genetics.

[122] Akashi H, Eyre-Walker A (1998). Translational selection and molecular evolution. Curr Opin Genet Develop.

[123] Baraket G, Abdelkrim AB, Salhi-Hannachi A (2015). tRNALeu intron (UAA) of *Ficus carica* L.: genetic diversity and evolutionary patterns. Genet Mol Res.

[124] Balakirev ES, Balakirev EI, Rodriguez-Trelles F, Ayala FJ (1999). Molecular evolution of two linked genes, *Est-6* and *Sod*, in *Drosophila melanogaster*. Genetics.

[125] Nachman NW, Crowell SL (2000). Contrasting evolutionary histories of two introns of the Duchenne muscular dystrophy gene, *Dmd*, in humans. Genetics.

[126] Gazave E, Marqués-Bonet T, Fernando O, Charlesworth B, Navarro A (2007). Patterns and rates of intron divergence between humans and chimpanzees. Genome Biol.

[127] Ding Y, Larson G, Rivas G, Lundberg C, Geller L, Ouyang C (2008). Strong signature of natural selection within an FHIT intron implicated in prostate cancer risk. PLoS One.

[128] Szabó JA, Szilágyi Á, Doleschall Z, Patócs A, Farkas H, Prohászka Z (2013). Both positive and negative selection pressures contribute to the polymorphism pattern of the duplicated human *CYP21A2* gene. PLoS One.

[129] Schaschl H, Huber S, Schaefer K, Windhager S, Wallner B, Fieder M (2015). Signatures of positive selection in the cis-regulatory sequences of the human oxytocin receptor (*OXTR*) and arginine vasopressin receptor 1a (*AVPR1A*) genes. BMC Evol Biol.

[130] Simonsen KL, Churchill GA, Aquadro CJ (1995). Properties of statistical tests of neutrality for DNA polymorphism data. Genetics.

[131] Jensen JD, Thornton KR, Aquadro CF (2008). Inferring selection in partially sequenced regions. Mol Biol Evol.

[132] Slatkin M, Hudson RR (1991). Pairwise comparisons of mitochondrial DNA sequences in stable and exponentially growing populations. Genetics.

[133] Benson DA, Cavanaugh M, Clark K, Karsch-Mizrachi I, Lipman DJ, Ostell J, Sayers EW (2013). GenBank. Nucleic Acids Res.

[134] Nei M (1987). Molecular Evolutionary Genetics.

[135] Watterson GA (1975). On the number of segregating sites in genetical models without recombination. Theor Popul Biol.

[136] Jukes TH, Cantor CR, Munro HM (1969). Evolution of protein molecules. Mammalian Protein Metabolism.

