# Supplementary material for: DNA polymorphism and selection at the bindin locus in three Strongylocentrotus sp. (Echinoidea)
Source: BMC Genet. 2016 May 12;17:66. doi: 10.1186/s12863-016-0374-5 (PMC4866015; doi:10.1186/s12863-016-0374-5)
Supplement: Additional file 1: — Text S1. PCR reactions. Text S2. GenBank bindin sequences. (DOC 68 kb) [file 12863_2016_374_MOESM1_ESM.doc]

**Additional file 1: Text S1.** PCR reactions

The PCR reactions were carried out in final volumes of 25 µl using TaKaRa Ex Taq™ in accordance with the manufacturer description (Takara Biotechnology Co., Ltd.). The reaction mixtures were placed in a DNA thermal cycler (Eppendorf, Mastercycler Gradient), incubated 5 min at 94° and subjected to 32 cycles of denaturation, annealing, and extension: 94° for 30 sec, 52° for 30 sec, and 72° for 1.0 min with a final 5-min extension period at 72° for the *COI* gene; 94° for 30 sec, 53° for 30 sec, and 72° for 1.5 min with a final 7-min extension period at 72° for the *bindin* gene. The obtained PCR products for the *bindin* gene were then cloned (TOPO TA cloning kit, Invitrogen, Calif.) and sequenced by the dideoxy chain-termination technique [1] using Dye Terminator chemistry and separated with the ABI PRISM 377 automated DNA sequencer (Perkin Elmer). For each clone, the sequences of both strands were determined, using overlapping internal primers spaced, on average, 500 nucleotides. At least two independent PCR amplifications were sequenced in both directions to prevent possible PCR or sequencing errors. The *COI* gene was sequenced directly (without cloning).

1. Sanger F, Nicklen S, Coulson AR (1977) DNA sequencing with chain terminating inhibitors. Proc. Natl. Acad. Sci. USA 75: 5463–5467.

**Additional file 1: Text S2.** GenBank *bindin* sequences

The additional *bindin* sequences of the genus *Strongylocentrotus* and close species were obtained from the GeneBank database, with the accession numbers: *S. purpuratus*, AF077309 and AF077310 [1]; *S. pallidus*, AF077313 and AF077314, AF133805 - AF133812 [1]; *S. droebachiensis*, AF077311 and AF077312, AF133794- AF133804 [1]; *Hemicentrotus pulcherrimus*, AF077318 and AF077319 [1]; *S. polyacanthus,* AF077316 and AF077317 [1].

**References**

1. Biermann CH. The molecular evolution of sperm bindin in six species of sea urchins (Echinoida: Strongylocentrotidae). Mol Biol Evol. 1998; 15: 1761-1771.
