# Supplementary material for: DNA polymorphism and selection at the bindin locus in three Strongylocentrotus sp. (Echinoidea)
Source: BMC Genet. 2016 May 12;17:66. doi: 10.1186/s12863-016-0374-5 (PMC4866015; doi:10.1186/s12863-016-0374-5)
Supplement: Additional file 2: Figure S1. — DNA polymorphism in the bindin repeat region (exon II) of Strongylocentrotus intermedius. Figure S2. DNA polymorphism in the bindin gene of Strongylocentrotus pallidus. The recombinant sequences (PAL_AF133806 and PAL_AF077313) are in bold. The exon—intron coordinates are: 1–237: exon I; 238–1189: intron; 1190–1543: exon II. Other comments see Fig. 1. Figure S3. DNA polymorphism in the bindin gene of Strongylocentrotus droebachiensis. The long 535-bp insertion has been deleted (see text for details). The exon - intron coordinates are: 1–237: exon I; 238–1189: intron; 1190–1516: exon II. Other comments see Fig. 1. Text S3. Repeat region. Text S4. Indel coordinates. (DOC 75 kb) [file 12863_2016_374_MOESM2_ESM.doc]

**Additional file 2: Figure S1**. DNA polymorphism in a repeat region of the *bindin* exon II of *Strongylocentrotus intermedius*

----------------------------------

11111112222222222222

15557888900468890022223344556

30291023414656896934780745169

************ ******* * *****

U-34 GTGCTACCAAACGAAGCCATCAAGGGCGA

U-16 ......T......................

G-43 .............................

U-35 .............................

G-42 .....................G.......

G-36 .............................

U-18 .............................

U-49 .............................

G-4 .C...........................

G-35 ............................C

G-38 .......................A.....

G-25 .............................

G-41 .............................

G-44 .............................

U-21 ...........................A.

U-30 A............................

U-48 .............................

U-7 .............................

G-45 ............................C

G-33 .............................

U-36 ............................C

G-30 ....G.......................C

G-23 ..AA.C.AGCCAAGCAAAGG..C.ATA.C

U-43 ..AA...AGCCAAGCAAAGG..C.ATA.C

U-8 ..AA...AGCCAAGCAAAGGA.C.ATA.C

----------------------------------

The specimens of *S. intermedius* are marked with letters “G” and “U”. For explanation of symbols, see legend to Figure 1

**Additional file 2: Figure S2.** DNA polymorphism in the *bindin* gene of *Strongylocentrotus pallidus*

---------------------------------------------------------------------------------------------------

1111111111 1 1 111

1112222 2 233333 344444 44 555666666 666777777777888888888 899 99990000012444 4 4 455

74880445 7 935579 914666 78 346114445 678000122445245667778 802 46691235927224 7 8 903

21134791 3 827804 788568 91 072010784 767589979125666021490 467 66948147476128 5 7 102

PAL_AF133808 **GGTAT**AT† ‡ CTACT▼2ACGTC▲2C‡ ATGCACAG‡ CCGCCTTTTAGACCTGTAGG‡ TA‡ GATCCCTACT**GCC▲3† † GCC**

***** ****** ******

PAL_AF077314 ........ . ....C. G..... .. ......... ..................... ... .............. . . ...

PAL_AF133807 ........ . ...... ...... .. ......... ..................... ... .............. . . ..A

PAL_EU700094 ........ . ....C. ...... .. ......... ..................... ... .............. . . ...

PAL_AF133805 ........ . ....C. ...... .. ......... ..................... ... .........A.... . . ...

**PAL_AF077313** .AATC.A▲1▼1G..... ...... .. .CT...... ..................... ... .............. . . ...

**PAL_AF133806** AAATC.A▲1▼1G.G..‡ .TA..† T▼3G...GT... ..................... ..▼6.............. . . ...

PAL_AF133809 .AATCGA▲1▼1GGG..‡ .T.... .. G...G..A▼4.TTT.G.A.C..TTGTAT.A. .C. .C.T..CC...GT. ▲4. CG.

PAL_AF133810 .AATC.A▲1▼1G.G..‡ .T...† .. G...G..A▼4.TTT.GGAAC...TGTAT.A. AC. .CAT..CC..AGT† . ▲5-G.

PAL_AF133811 .AATC.A▲1▼1G.G... .T.CT† .. G..TG.TA▼4TTTTTG.A.CT..TGTATCA▼5.C. AC.TT.CCT..GT† . ▲5-G.

PAL_AF133812 .AATC.A▲1▼1G.GT.‡ .T.CT† .. G..TG.TA▼4.TTTTG.A.C.G.TGTATCA. .C. .C.TTACCT..GT† . . CG.

---------------------------------------------------------------------------------------------------

PAL = *S. pallidus*. The geographic origins of the *S. pallidus* *bindin* alleles can be found in the Appendix of [1]. For explanation of symbols, see legend to Figure 1.

**References**

1. Marks J.A., Biermann C.H., Eanes W.F., Kryvi H. Sperm polymorphism within the sea urchin *Strongylocentrotus droebachiensis*: divergence between Pacific and Atlantic oceans. Biol. Bull. 2008. 215(2):115-125.

**Additional file 2: Figure S3**. DNA polymorphism in the *bindin* gene of *Strongylocentrotus droebachiensis*

------------------------------------------------------------------

111111 11111111 1

11223 3344 4 4 56 66 67777778 8889000011 12233444 4

4478891 7813 5 9 70 34 61134893 5796011457 95808122 6

8985965 4055 7 0 57 83 26799297 8869816938 72907757 4

DRO_AF133800 **AGGG**TG‡ TCT‡ ‡ T A† C‡ TACGGGAT GTATTGTCTT **ACACTGG‡ C**

******* ***** ****** *****

DRO_AF133801 ....... .... . . .. .. ........ .......... ........ .

DRO_AF133802 T...... .... . . .. .. ........ .......... ........ .

DRO_AF133803 T...... .... . . .. .. ........ .......... ........ .

DRO_EU663624 ......▼1..G. . . .. .. ........ T.....C... ........ .

DRO_EU663623 ....... C... . . .. .. ........ ....C...C. ........ .

DRO_EU663625 .A..G.. .... . A .. .▼4..A...G. .AC..T.T.C .T..CTA▼5.

DRO_AF133799 ....G.. .... . A .. .▼4..A...G. .AC..T.T.. .T..CTA▼5G

DRO_AF133804 ....G.. .... ▼3A .. .▼4.CA...G. .AC....T.. .T..CTA▼5G

DRO_AF077312 ....... ...▼2. A .. .. .CA...GA .AC....T.. ....C.A▼5G

DRO_AF133796 ..AA.A. .G.. . A .. .. .CA.C.G. .AC....T.. C.C.C.A▼5G

DRO_AF133797 ..AA... .T.. . A .. .. .CA.C.G. .AC....T.. C.C.C.A▼5G

DRO_AF133795 ..AA... .T.. . A .. T. .CA.C.G. .ACA...T.. C.C.C.A▼5G

DRO_AF133794 ..AA... .T.. . A .. .. ACA.C.G. .AC....T.. C.CTC.A▼5G

DRO_AF077311 ..AA... .T.. . A C▲1.. .CAACTG. .AC....T.. C.C.C.A▼5G

DRO_AF133798 ..AA... .T.. . A C▲1.. .CAACTG. .AC....T.. C.C.C.A▼5G

------------------------------------------------------------------

DRO = *S. droebachiensis*. The geographic origins of the *S. droebachiensis* *bindin* alleles can be found in the Appendix of [1]. For explanation of symbols, see legend to Figure 1.

**References**

1. Marks J.A., Biermann C.H., Eanes W.F., Kryvi H. Sperm polymorphism within the sea urchin *Strongylocentrotus droebachiensis*: divergence between Pacific and Atlantic oceans. Biol. Bull. 2008. 215(2):115-125.

**Additional file 2: Text S3.** Repeat region

The *bindin* gene contains a 21-bp repeat motif [1, 2] in the 3’ region of exon II. The region is highly variable in number of repeats, even within a single species: from two in *S. franciscanus* [2] to 6-9 in *S. droebachiensis*, and 11-12 in *S. pallidus* [1]. There are 13 variable repeats in *S. intermedius* (273 bp in coordinates 1513 – 1785 for the alignment of 25 *bindin* nucleotide sequences). We detected 29 polymorphic sites within the region (9 singletons and 20 parsimony informative sites; Additional file 2: Figure S3), which are mostly nonsynonymous (25 out of 29 totally; Additional file 2: Figure S3) as it was previously detected in other sea urchin species [1]. The level of nucleotide diversity is high: π = 0.0195±0.0037, which is three times more variable than the whole *bindin* gene excluding repeat region (π = 0.0060±0.0010). The difference is mostly due to replacement substitutions, which are 10 times more frequent in the repeat region than in the rest of the *bindin* coding region: π = 0.0216 versus 0.0021 (Table 1). The synonymous variability is similar for both, the repeat region (π_syn_ = 0.0111) and the rest of the gene (π_syn_ = 0.0076) (Table 1).

The region is not a discriminate marker for the *S. intermedius* morphological forms: the sequences from the two forms are intermingled with no evidence of discrete species heterogeneity (*F*_st_ = - 0.0438, *P* = 0.5480; total sequence divergence between the forms *D*_xy_ = 0.0193±0.0037). Interestingly, the total sequence divergence between the forms (*D*_xy_) based on the repeat region is three times greater than the *D*_xy_ based on the rest of the gene excluding the repeat region (*D*_xy_ = 0.0193±0.0037 versus 0.0064±0.0011). We suggest that this difference is sampling error due to the highly asymmetrical haplotype distribution for both, the repeat region and the rest of the gene (Additional file 2: Figure S3; Figure 1). In the repeat region, two out of three divergent haplotypes are represented by the U form (Additional file 2: Figure S3), while for the rest of the gene, three out of five divergent haplotypes are represented by the U form (Figure 1). This difference in the number of divergent haplotypes is essential for the *D*_xy_ calculation taking into account the very short length of the repeat region (273 bp). The repeat region was excluded from the analysis of diversity and selection in *S. intermedius* because orthology/paralogy relationships are uncertain in these sequences.

**References**

1. Biermann C.H. The molecular evolution of sperm bindin in six species of sea urchins (Echinoida: Strongylocentrotidae). Mol Biol Evol. 1998; 15: 1761-1771.

2. Minor J.E., Fromson D.R., Britten R.J., Davidson E.H. Comparison of the bindin proteins of *Strongylocentrotus franciscanus*, *S. purpuratus*, and *Lytechinus variegatus*: sequences involved in the species specificity of fertilization. Mol. Biol. Evol. 1991; 8: 781-795.

**Additional file 2: Text S4.** Indel coordinates

Coordinates of indels in the *bindin* gene regions of *Strongylocentrotus intermedius* (A), *S. pallidus* (B), and *S. droebachiensis* (C). The repeat region within exon II was excluded from the analysis (see text for details).

**A.** Coordinates of indels in the *bindin* gene of *Strongylocentrotus intermedius*

Position 457: ▲1, a single nucleotide deletion of A; position 583: ▲2, a 16-bp deletion of CCGTGACTCGGGTCTG; position 775: ▲3, a single nucleotide deletion of T; position 822: ▲4, a 41-bp deletion of TTGCCGTTACCAAGGTTTTGCCTGAATGAATTTTTTCTTTA; position 857: ▲5, a single nucleotide deletion of T; position 858: ▼1, a single nucleotide insertion of T; position 888: ▼2, a single nucleotide insertion of A; position 894: ▲6, a single nucleotide deletion of T; position 953: ▼3, a 2-bp insertion of TA; position 1046: ▼4, a 2-bp insertion of TA; position 1061: ▲7, a 17-bp deletion of AGTGCTCAGTTTTTATC.

**B.** Coordinates of indels in the *bindin* gene of *Strongylocentrotus pallidus*

Position 251 (266 - general file): a 5-bp deletion (251-255); TTATT; position 273 (277 - general file) a 2-bp insertion (273-274); GT (AT); position 394 (414 - general file) a single nucleotide insertion of T; position 468 (497 - general file) a single nucleotide deletion of C; position 481 (510 - general file) a 2-bp insertion (481-482) of TA; position 654 (693 - general file) a 2-5 bp insertion of A; position 880 (930 - general file) a single nucleotide insertion of A; position 927 (985 - general file) a single nucleotide insertion of C; position 1448 (1520 - general file) a 27 bp deletion of GGTGGCGCAGGGGGCATGGGTATCGGC; position 1448 (1547 - general file) a 3-bp deletion of GGTGGCGCAGGGGGCATGGGTATCGGCGGA; position 1487 (1559 - general file) a 12 bp deletion of GGGGCCATGATG.

**C.** Coordinates of indels in the *bindin* gene of *Strongylocentrotus droebachiensis*.

Position 315 (335 – general file): ▼1, a 2-bp insertion (315-316) of TA; position 435 (457 – general file): ▼2, a 2-bp insertion (435-436) of AT; position 457 (479 – general file): ▼3, a 7-bp insertion (457-463) of TCACTAC; position 607 (637 – general file): ▲1, a 20-bp deletion (607-626) of ATAAGAGTCGGCTTAAGGAA; position 643 (675 – general file): ▼4, a 2-bp insertion (643-644) of AT; position 1427 (1499 – general file): ▼5, a 9-bp insertion (1427-1435) of GCACCAGGC.
