# Supplementary material for: DNA polymorphism and selection at the bindin locus in three Strongylocentrotus sp. (Echinoidea)
Source: BMC Genet. 2016 May 12;17:66. doi: 10.1186/s12863-016-0374-5 (PMC4866015; doi:10.1186/s12863-016-0374-5)
Supplement: Additional file 3: Table S1. — Recombination estimates with RDP3. Table S2. The bindin gene recombination estimates (ρ). (DOC 45 kb) [file 12863_2016_374_MOESM3_ESM.doc]

**Additional file 3: Table S1.** Recombination estimates with RDP3 in the sea urchins

| Method | Reference | Species | Average *P* - value |
| --- | --- | --- | --- |
| LARD | [1] | *S. intermedius* | 5.860 × 10^-03^ |
|  |  | *S. pallidus* | 1.822 × 10^-07^ |
| GENECONV | [2] | *S. intermedius* | 1.573 × 10^-02^ |
|  |  | *S. pallidus* | 1.612 × 10^-02^ |
| BOOTSCAN | [3, 4] | *S. intermedius* | 1.302 × 10^-2^ |
|  |  | *S. pallidus* | 1.024 × 10^-03^ |
| MAXCHI | [5] | *S. intermedius* | NS |
|  |  | *S. pallidus* | 5.987 × 10^-05^ |
| CHIMAERA | [6] | *S. intermedius* | NS |
|  |  | *S. pallidus* | 3.399 × 10^-03^ |
| SiScan | [7] | *S. intermedius* | NS |
|  |  | *S. pallidus* | 6.620 × 10^-09^ |
| 3Seq | [8] | *S. intermedius* | 7.669 × 10^-02^ |
|  |  | *S. pallidus* | 2.816 × 10^-05^ |

Detection of recombination in the *bindin* gene by seven different methods implemented in RDP3 [9] in sea urchin *Strongylocentrotus intermedius* and *S. pallidus*. The parental and recombinant sequences were determined using the VisRD method [10], modified version of PHYLPRO [11], and EEEP [12] also implemented in RDP3 (default settings). NS: not significant signal of recombination.

**References**

1. Holmes EC, Worobey M, Rambaut A. Phylogenetic evidence for recombination in dengue virus. Mol Biol and Evol. 1999; 16: 405-409.

2. Padidam M, Sawyer S, Fauquet CM. Possible emergence of newgeminiviruses by frequent recombination. Virology. 1999; 265: 218–225.

3. Martin DP, Posada D, Crandall KA, Williamson C. A modified bootscan algorithm for automated identification of recombinant sequences and recombination breakpoints. Aids Res Hum Retrovir. 2005; 21: 98–102.

4. Bredell H, Martin DP, Van Harmelen J, Varsani A, Sheppard HW, Donovan R, et al. Team HS: HIV type 1 subtype C gag and nef diversity in southern Africa. Aids Res Hum Retrovir. 2007; 23: 477–481.

5. Smith JM: Analyzing the mosaic structure of genes. J Mol Evol. 1992; 34: 126–129.

6. Posada D, Crandall KA. Evaluation of methods for detecting recombination from DNA sequences: computer simulations. Proc Natl Acad Sci USA. 2001; 98: 13757–13762.

7. Gibbs MJ, Armstrong JS, Gibbs AJ. Sister-Scanning: a Monte Carlo procedure for assessing signals in recombinant sequences. Bioinformatics, 2002; 16: 573-582.

8. Boni MF, Posada D, Feldman MW. An exact nonparametric method for inferring mosaic structure in sequence triplets. Genetics. 2007; 176: 1035-1047.

9. Martin DP, Lemey P, Lott M, Moulton V, Posada D, Lefeuvre P. RDP3: a flexible and fast computer program for analyzing recombination. Bioinformatics. 2010; 26: 2462-2463.

10. Lemey P, Lott M, Martin DP, Moulton V. Identifying recombinants in human and primate immunodeficiency virus sequence alignments using quartet scanning. BMC Bioinformatics. 2009;10: 126.

11. Weiller GF. Phylogenetic profiles: a graphical method for detecting genetic recombinations in homologous sequences. Mol Biol Evol. 1998; 15: 326–335.

12. Beiko RG, Hamilton N. Phylogenetic identification of lateral genetic transfer events. BMC Evol Biol. 2006; 6: 15.

**Additional file 3: Table S2.** The *bindin* gene recombination estimates (*ρ*)

|  | All sites |  | Informative sites only |  |
| --- | --- | --- | --- | --- |
|  | Per gene Per site |  | Per gene Per site |  |
| INT  PAL  DRO | 7.107 0.0047  0.701 0.0005  0 0 |  | 9.309 0.0061  0.701 0.0005  1.602 0.0011 |  |

_________________________________________________________

The population recombination rate, *ρ*, is 4*N_e_r* (*N_e_* is the effective population size and *r* is the recombination rate / nucleotide site / generation), obtained by the method of McVean et al. [1]. All sites, as well as informative sites, are included in the recombination analysis. The indels are excluded from the analyzed sequences. INT = *S. intermedius*, DRO = *S.* *droebachiensis*, PAL = *S. pallidus*.

**Reference**

1. McVean G, Awadalla P, Fearnhead P. A coalescent-based method for detecting and estimating recombination from gene sequences. Genetics. 2002; 160: 1231–1241.
