# Supplementary figures and images for: DNA polymorphism and selection at the bindin locus in three Strongylocentrotus sp. (Echinoidea)
Source: BMC Genet. 2016 May 12;17:66. doi: 10.1186/s12863-016-0374-5 (PMC4866015; doi:10.1186/s12863-016-0374-5)

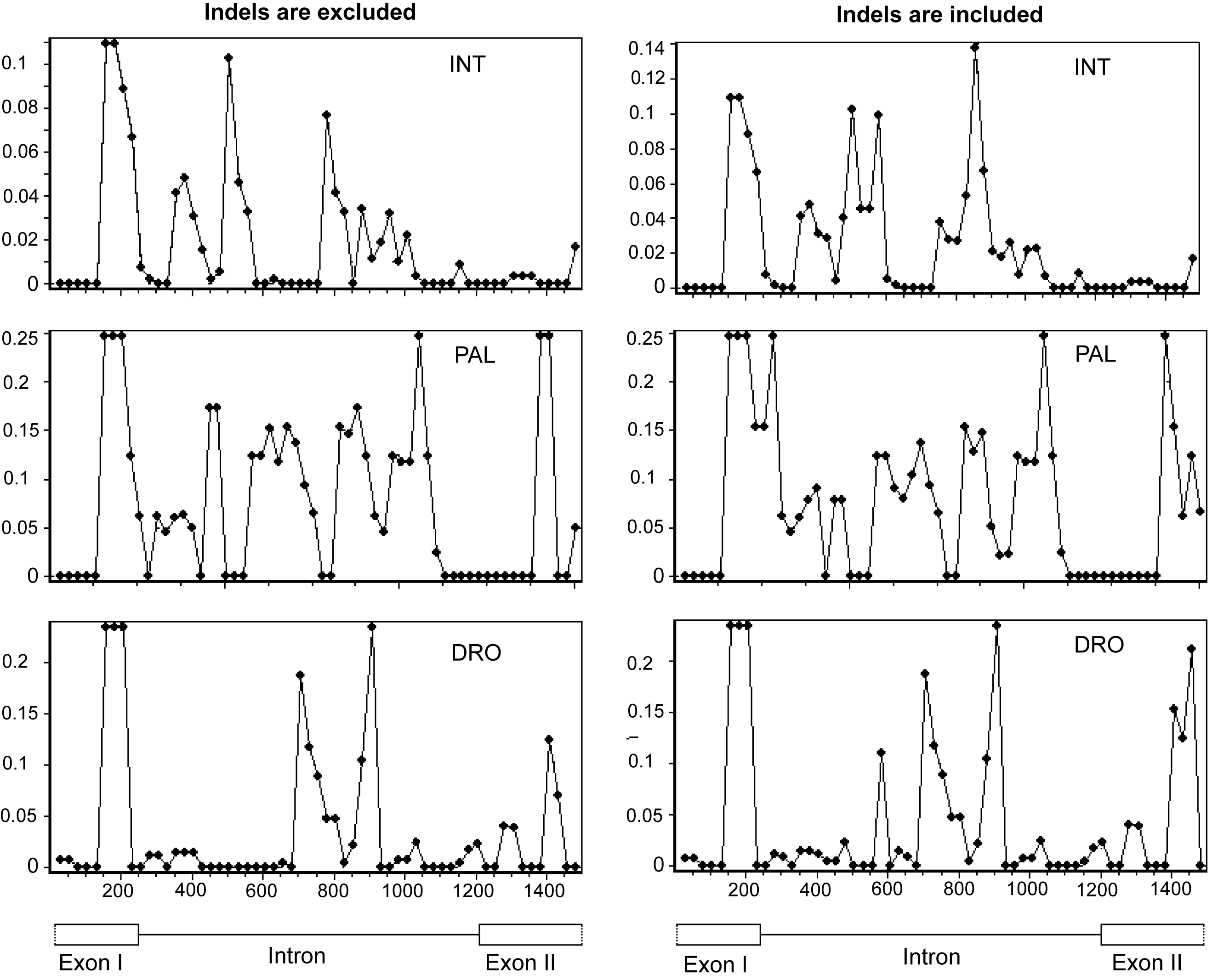

Supplement: Additional file 4: Figure S4. — Sliding window plots of linkage disequilibrium (measured by D) along the bindin gene region in S. intermedius (INT), S. pallidus (PAL), and S. droebachiensis (DRO). Indels are excluded (left side) or included (right side). Window sizes are 60 nucleotides with 25-nucleotide increments. A schematic representation of the bindin gene is displayed at the bottom. (TIF 584 kb) [file 12863_2016_374_MOESM4_ESM.tif]

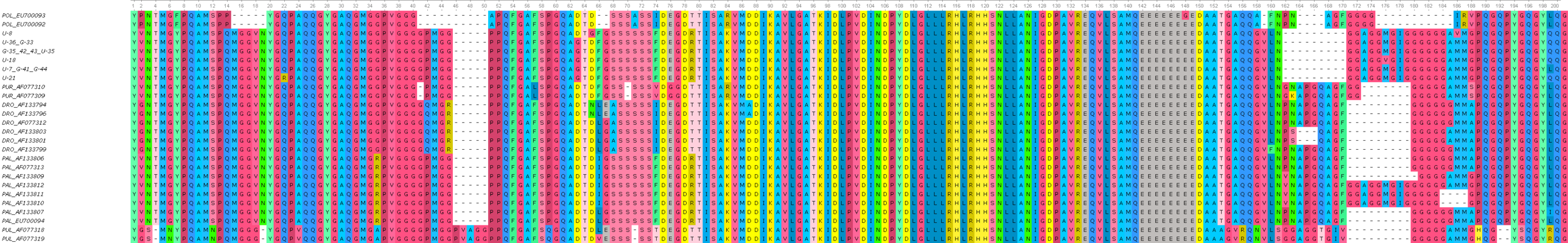

Supplement: Additional file 5: Figure S5. — Bindin amino acid alignment. INT = S. intermedius, DRO = S. droebachiensis, PAL = S. pallidus; POL = S. polyacanthus; PUR = S. purpuratus; PUL = Hemicentrotus pulcherrimus. (PNG 178 kb) [file 12863_2016_374_MOESM5_ESM.png]
